# Global research trends and hotspots on human intestinal fungi and health: a bibliometric visualization study

**DOI:** 10.3389/fcimb.2024.1460570

**Published:** 2024-10-17

**Authors:** Ming Gong, Huiying Yu, Hong Qu, Zhexian Li, Di Liu, Xin Zhao

**Affiliations:** ^1^ Department of Cardiology, The Second Hospital of Dalian Medical University, Dalian, China; ^2^ Laboratory of Basic Medicine, General Hospital of Northern Theater Command, Shenyang, China; ^3^ Bidding and Procurement Office, The Second Hospital of Dalian Medical University, Dalian, China; ^4^ Dalian Medical University, Dalian, China; ^5^ First Clinical Faculty, Liaoning University of Traditional Chinese Medicine, Shenyang, China

**Keywords:** bibliometric, fungal microbiota, inflammatory bowel disease, CiteSpace, VOSviewer

## Abstract

**Background:**

This article employs bibliometric methods and visual maps to delineate the research background, collaborative relationships, hotspots, and trends in the study of gut fungi in human diseases and health.

**Methods:**

Publications related to human gut fungi were retrieved from the Web of Science Core Collection. VOSviewer, CiteSpace, R software and Microsoft Excel were employed to generate visual representations illustrating the contributions made by countries/regions, authors, organizations, and journals. Employing VOSviewer and CiteSpace, we conducted a comprehensive analysis of the retrieved publications, revealing underlying tendencies, research hotspots, and intricate knowledge networks.

**Results:**

This study analyzed a total of 3,954 publications. The United States ranks first in the number of published papers and has the highest number of citations and h-index. Mostafa S Elshahed is the most prolific author. The University of California System is the institution that published the most papers. Frontiers In Microbiology is the journal with the largest number of publications. Three frequently co-cited references have experienced a citation burst lasting until 2024.

**Conclusion:**

Advancements in sequencing technologies have intensified research into human gut fungi and their health implications, shifting the research focus from gut fungal infections towards microbiome science. Inflammatory bowel diseases and Candida albicans have emerged as pivotal areas of interest in this endeavor. Through this study, we have gained a deeper insight into global trends and frontier hotspots within this field, thereby enhancing our understanding of the intricate relationship between gut fungi and human health.

## 1 Introduction

The fungal microbiota plays a crucial role in the intricate, multikingdom microbial community residing within the mammalian gastrointestinal tract. Historically, microbiome research has been predominantly bacteria-oriented (Manos, 2022), with fungi receiving less attention (Paterson et al., 2017). Although fungal microbiota constitute a relatively minor fraction of the gut microbiome and have historically received less attention compared to their more abundant bacterial counterparts, their significance in both provoking and defending against pathologies is gradually being recognized (Pappas et al., 2018). Recent advancements in sequencing technologies have furnished us with sophisticated tools to comprehensively characterize the fungal component of the gut microbiome (Soverini et al., 2019). Emerging evidence underscores the pivotal role of the gut mycobiome as a potential reservoir harboring opportunistic pathogens that may contribute to the pathogenesis of inflammatory bowel disease (IBD) (Li et al., 2014; Liguori et al., 2016), graft-versus-host disease (GVHD) (Zhang F. et al., 2021), gastrointestinal cancer (Luan et al., 2015; Gao et al., 2017; Coker et al., 2019), as well as a multitude of other diseases. These discoveries underscore the intricate interplay between the gut mycobiome and human health, highlighting new avenues for disease prevention, diagnosis, and potential therapeutic interventions.

Bibliometric is an innovative approach for summarizing research advancements and pinpointing emerging trends or focal points within a research domain through the creation of informative graphics. This technique leverages the power of mathematics and statistics to analyze diverse knowledge carriers, employing software tools to visualize the distribution, connections, and evolving patterns among countries, institutions, authors, journals, and research areas (Cancino et al., 2017). By providing insights into these hotspots and trends, bibliometrics offers valuable predictions for the future development of a particular field, enabling researchers and policymakers to make informed decisions based on evidence-based analytics (Ma et al., 2022). This methodology has gained widespread recognition as an effective research framework for evaluating impact and evidence, bolstered by the expanding availability of research data through repositories such as Web of Science (WOS) (Chen et al., 2020). Although bibliometric studies on human intestinal fungi remain scarce, this paper aims to explore the existing literature in this field over the past 24 years through bibliometric analysis. Consequently, we conducted a systematic investigation to assess the current research landscape and identify key areas of interest and research hotspots.

## 2 Materials and methods

### 2.1 Data collection and search scheme

**Figure 1:**
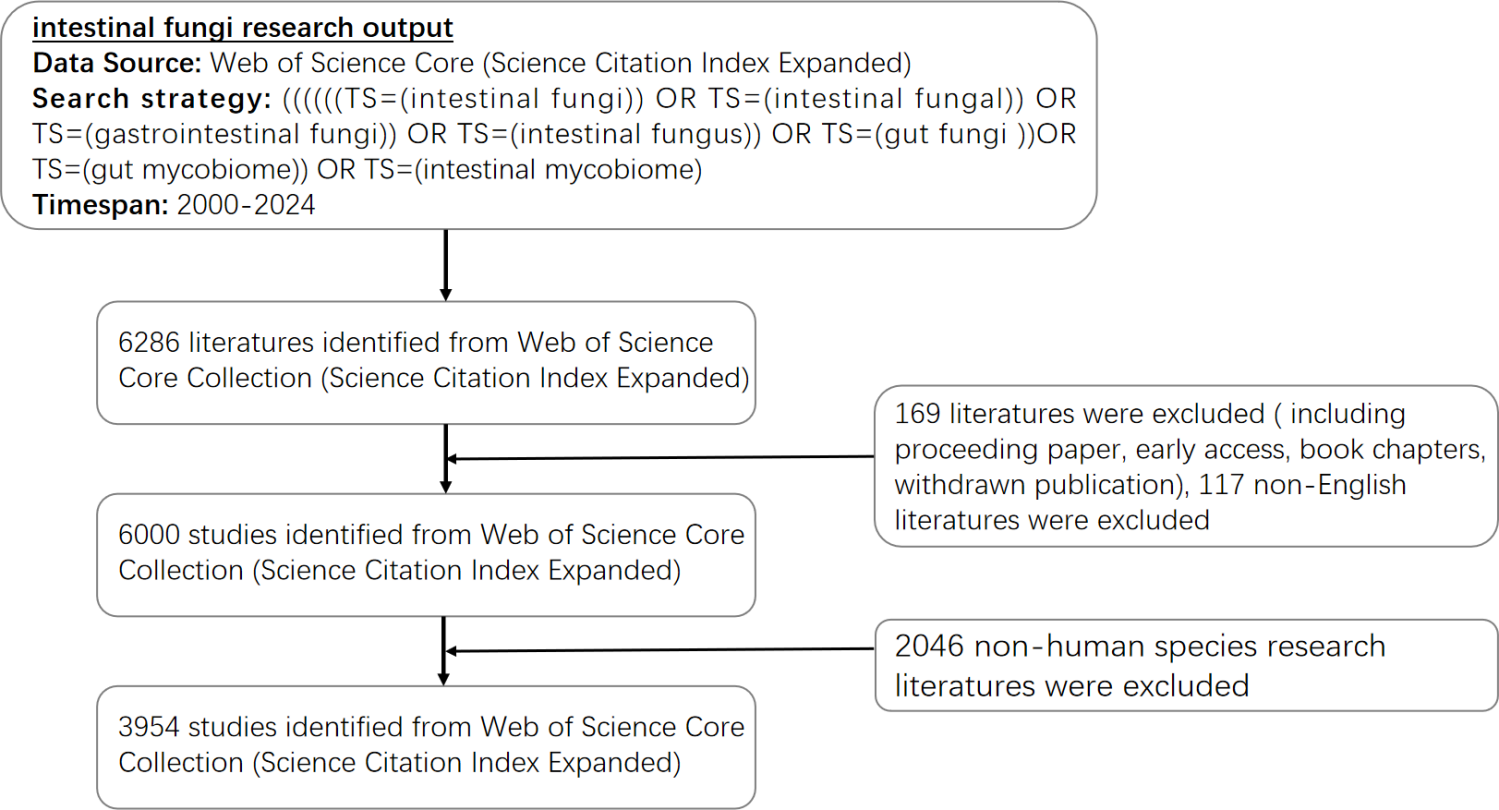
A diagram depicted the sequential evaluation and selection steps. The study encompassed English-language articles published between 2000 and 2024, with the removal of any duplicate publications.

The WoSCC, renowned for its vast interdisciplinary coverage, encompasses an impressive collection of over 170 million articles spanning across more than 250 academic disciplines. Notably, this database harbors a substantial quantity of literature directly relevant to our study. Given its widespread utilization and esteemed reputation within scientometric research, the WoSCC serves as a robust foundation for delving into and analyzing the progression of gut fungi research within our field (Feng et al., 2023). Consequently, it was chosen as the preferred database to acquire global academic information for bibliometric analysis, building upon a foundation of previous publications (Song et al., 2023). All the published literature was retrieved from the SCI-Expanded database, with a search period spanning from 1 January 2000 to 19 September 2024. Data retrieval was set for September 19, which meant that the most recent studies were included as much as possible. The search strategy employed in the WOSCC database followed the method outlined below: ((((((TS=(intestinal fungi)) OR TS=(intestinal fungal)) OR TS=(gastrointestinal fungi)) OR TS=(intestinal fungus)) OR TS=(gut fungi))OR TS=(gut mycobiome)) OR TS=(intestinal mycobiome). We filtered out non-English literature and limited our analysis to articles and reviews, ultimately yielding 6,000 eligible articles. Subsequently, we excluded research literature related to gut fungi in non-human species such as veterinary medicine and botany through Web of Sciences Categories and manual screening. Finally, 3954 papers were acquired (as illustrated in **Figure 1**).

Given the significance of the Scopus database in scientific research, the research team of this paper has included relevant analytical information and charts from the Scopus database in the **Supplementary Materials**, aiming to provide readers with a broader, more comprehensive, and rigorous understanding of the research landscape in the field of gut mycobiome.

### 2.2 Data gathering and refining

The initial dataset, generated using the designated methodology, encompassed crucial metrics such as the total number of papers, citation count, H-index, as well as pertinent datapertaining to affiliations, authors, journals, and geographical information. We excluded any items with incorrect spellings and removed duplicate authors to ensure the accuracy of our analysis. It is noteworthy that cited references often exist in numerous versions or variations. Moreover, it is not uncommon for multiple authors to share identical name abbreviations, and the formatting of cited journals often varies significantly. These factors can potentially introduce inaccuracies into the analysis, which should be taken into consideration. Therefore, we manually organized the data to prevent duplication. By employing a thesaurus file, we effectively addressed the challenges posed by incorrectly spelled elements and duplicates. For example, we substitute “Taiwan”/”Hong Kong”/”People R China” with the term “China”. After refining the dataset, we conducted a bibliometric analysis using VOSviewer (version 1.6.19.0), CiteSpace (version 6.2. R4), and the “bibliometrix package 4.1.2” of R software (version 4.3.1).

### 2.3 Bibliometric analysis and visualization

The eligible studies were characterized by the inherent functionality of WOSCC. Additionally, the H-index was computed to assess the impact of scientific research, which represents the number of scholars who have published H papers and received at least H citations (Hu et al., 2022). The g-index represents the highest number of papers that have achieved citation counts equal to or exceeding the h-index of a scholar (Abbas, 2012). Furthermore, the impact factor (IF) from the latest Journal Citation Reports can serve as an metric to indicate the value of an article (Jones et al., 2011; Roldan-Valadez et al., 2019).

Different software tools were utilized for performing bibliometric analysis in the research. For the visualization and creation of bibliometric networks, we used the VOSviewer software, which was developed by Leiden University in the Netherlands. This software facilitated a 4comprehensive examination of bibliographic coupling, co-citation, co-occurrence, and international collaborations, enabling a deeper understanding of the relationships and patterns within the bibliometric data (van Eck and Waltman, 2017). Utilizing its ability to simultaneously map and identify clusters within a network, the software was employed alongside clustering methods to segment the networks into distinct clusters. This approach considered the strength of connections between nodes, ensuring a more accurate and nuanced understanding of the network structure. Furthermore, we utilized the “bibliometrix” package based on R4.1.2 to generate visualizations that depict the relationships among various journals. Additionally, in the analysis, we employed CiteSpace, a software tool developed by Professor [ (Chen and CiteSpace, 2006), to generate dual-map overlays for journals, conduct a cited keywords analysis, and identify references with notable citation bursts, ultimately revealing valuable insights into evolving patterns and trends in the field of study.

Both VOSviewer and CiteSpace are utilized for bibliometric analysis, sharing the aim of visually representing literature networks, collaborative relationships, and thematic trends. VOSviewer focuses on co-occurrence analysis and provides customizable network visualizations, enabling a deeper understanding of keyword associations and collaborations. CiteSpace emphasizes temporal analysis and citation networks, revealing research history and impact. By utilizing both VOSviewer and CiteSpace in this study, we aim to gain a more comprehensive understanding of the research landscape. Furthermore, online tools facilitate the creation of chord diagrams depicting relationships among countries, providing a visual representation of their interactions. These chord diagrams proportionally divide the circle based on country distribution and utilize chord segments of varying widths to visually represent the relationships and connections between interrelated nations, with the width serving as an indicator of the intensity of these associations.

## 3 Results

### 3.1 Annual publications and trend

**Figure 2:**
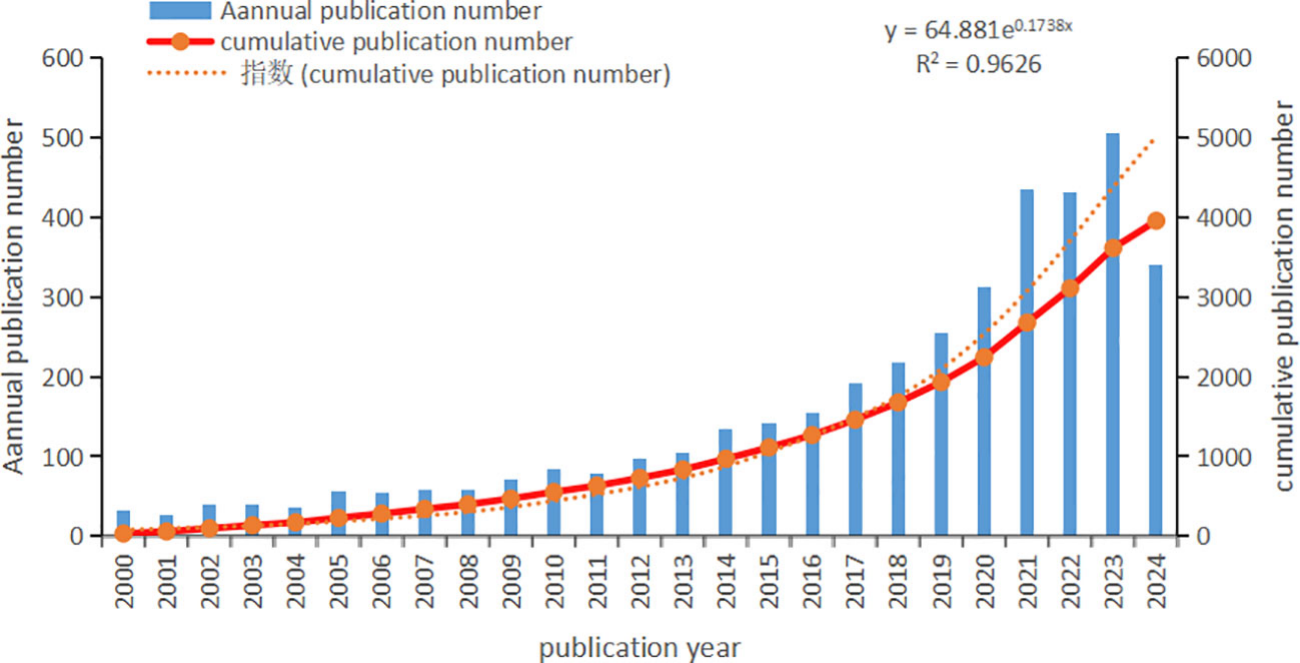
Intestinal fungal related Publication volume overview. The annual publication number and cumulative publication number during 2000 and 2024.

Based on our search strategy, we gathered a total of 3,954 literature pieces spanning from January 1st, 2000, to September 19th, 2024, with **Figure 2** depicting the yearly publication outcomes related to intestinal fungi. There were 2,882 articles and 1,072 review articles. The interest in gastrointestinal fungi has been on a significant upward trajectory over the past 20 years. In 2000, there were merely 33 publications dealing with intestinal fungi, gradually growing to 97 in 2012, and ultimately peaking at a record high of 505 in 2023. Notably, since 2020, there has been a substantial surge in the volume of published articles. The total number of global publications on this topic in 2023 is approximately 14.75 times higher than that in 2000, marking a significant milestone. The rapid increase in publication numbers reflects the growing interest among scholars in this area, indicating active research and the thriving development of theories related to intestinal fungi.

### 3.2 Contribution of countries/regions

Global attention has been drawn to research on intestinal fungi, as ongoing studies span across 117 countries and regions worldwide. The ranking of the top 10 countries/regions was determined by their publication count, along with their citation frequency, H-index, and centrality. The centrality metric highlights the importance of each country/region within the overall research network (as seen in **Table 1**). The centrality metric quantifies the strength of connections among nodes within a network, thereby indicating their significance and influence. Notably, the top 10 countries/regions that have contributed over 3000 papers on intestinal fungi are predominantly situated in Asia, Europe, and North America. The United States leads the rankings with 1140 publications (28.83% share), followed closely by China with 876 counts (22.15%) and Germany rounding out the top three with 284 counts (7.12%). The combined output of academic publications from China and the United States comprises a substantial 50.99% of the global total, exceeding that of any other country by a significant margin.

**Table 1:** The top 10 countries with the most publications on gut fungi.

Rank	Countries/ regions	counts	Citing article	citations	Average citations	H-index	Centrality
1	UNITED STATES	1140	23155	50674	44.45	218	0.19
2	CHINA	876	23050	16440	18.77	108	0.13
3	GERMANY	284	6246	7841	27.61	115	0.14
4	ITALY	231	5617	8771	37.97	107	0.06
5	FRANCE	212	5026	8316	39.23	119	0.15
6	INDIA	192	4635	3724	19.40	70	0.08
7	CANADA	188	3976	4923	26.19	84	0.02
8	ENGLAND	188	5057	5468	29.09	75	0.12
9	BRAZIL	164	4112	2619	15.97	63	0.12
10	SPAIN	149	4063	2784	18.68	84	0.04

The United States leads in citation frequency, boasting an impressive total of 50,674 citations, while China (16,440) and Germany (7,841) follow suit. Notably, the United States exhibits a strong impact with a citation count of 44.45 per publication, underscoring its prominent position in the academic sphere. In comparison, China exhibited a high citation count, yet its citation-to-publication ratio was relatively low when compared to the leading 10 countries, with a value of 18.77. It is worth noting that the citation/publication ratios in the Italy and France are as high as 37.97 and 39.23, respectively, indicating a high quality of their published papers, despite their relatively low number of publications. Regarding the H-index, the United States emerged as the leading country with a score of 218, followed by France and Germany, which achieved H-indices of 119 and 115, respectively.

**Figure 3:**
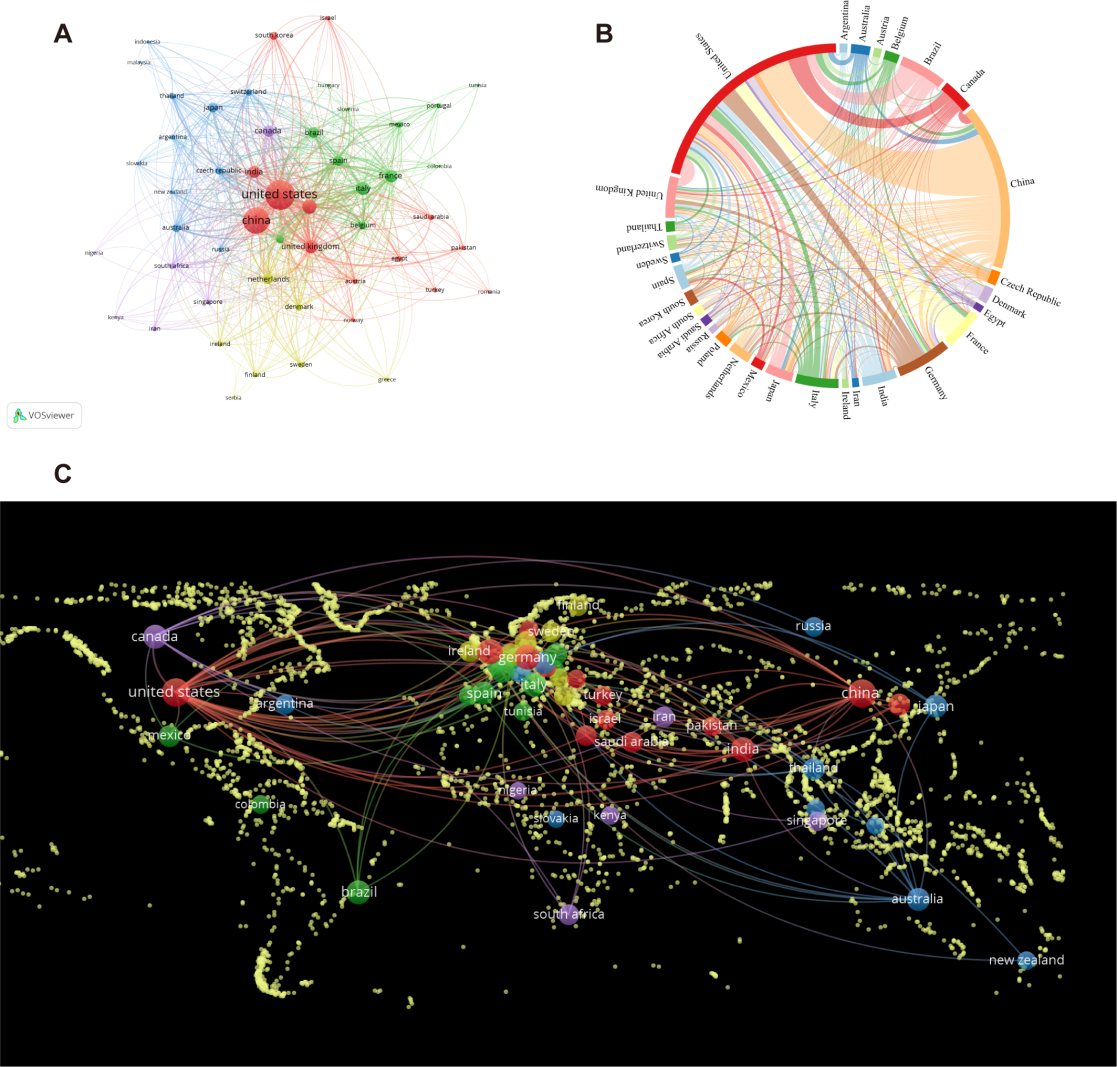
The involvement of various countries in intestinal fungal research. **(A)** intestinal fungal research collaboration map across countries. Countries are represented by circles, while lines illustrate their collaborations. The weight corresponds to the publication count, line thickness denotes the strength of collaboration, and distinct colors highlight clusters. **(B)** A chord diagram assessing the global collaboration among clusters. **(C)** World map illustrating the density of cooperation among countries.

The analysis of **Figures 3A** offers insights into the collaborative patterns among the top 50 countries, as determined by their publication output. The results indicate that VOSviewer can categorize these countries into five distinct clusters, reflecting varying degrees of collaboration among them. The clusters are visually distinguished through the use of varying colors, and **Figure 3C** visually represents the collaborative ties among the previously mentioned countries via a world map. Furthermore, a chord diagram is utilized to depict the top 30 countries, with each country assigned a unique color for enhanced clarity. The entire circle is divided proportionally based on each country’s representation, the wider the chord segments, the stronger the collaborative relationships among these countries are (shown in **Figure 3B**). In summary, collaboration primarily centered around China and the United States, with Germany, Canada, India, and Denmark also being significant collaborators. However, a more limited level of collaboration was observed with other countries.

### 3.3 Contributions of institutions

The analysis of research institutions offers insights into the global distribution of intestinal fungal research, enabling the identification of potential collaboration partners. In **Table 2**, you can find the top 10 institutions, with University of California System being the most productive (158, 4.20%), followed by the Institut National de la Sante et de la Recherche Medicale (Inserm) (85, 2.14%) and Chinese Academy of Sciences (83, 2.10%). France held a prominent position in the top 10 institutions, accounting for the majority (4 out of 10) and representing 6.73% of the overall papers published. The Centre National de la Recherche Scientifique (CNRS) stands apart with the highest average citations, at 31.03, closely followed by Harvard University and Chinese Academy of Sciences, which boast average citations of 26.57 and 23.95, respectively. The research institution with the highest H-index is the University of California System (63), followed by Harvard University (53), and University Of California San Diego (48) in the top three. Our analysis shows that among the top ten universities with the highest publication volume in this field, American universities have performed outstandingly in terms of publishing quality.

**Table 2:** The top 10 institutions conducting intestinal fungal by volume.

Rank	Institutions	Country	Counts	Citations	Average citations	H-index	Centrality
1	University of California System	United States	158	2853	18.06	63	0.2
2	Institut National de la Sante et de la Recherche Medicale (Inserm)	France	85	1666	19.60	34	0.06
3	Chinese Academy of Sciences	China	83	1988	23.95	24	0.08
4	Centre National de la Recherche Scientifique (CNRS)	France	62	1924	31.03	34	0.06
5	Harvard University	United States	61	1621	26.57	53	0.07
6	Universite Paris Cite	France	60	1329	22.15	30	0.01
7	INRAE	France	59	1335	22.63	30	0.01
8	University Of California San Diego	United States	58	694	11.97	48	0.02
9	Cornell University	United States	51	733	14.37	42	0.06
10	Czech Academy Of Sciences	Czech Republic	50	546	10.92	23	0.06

**Figure 4:**
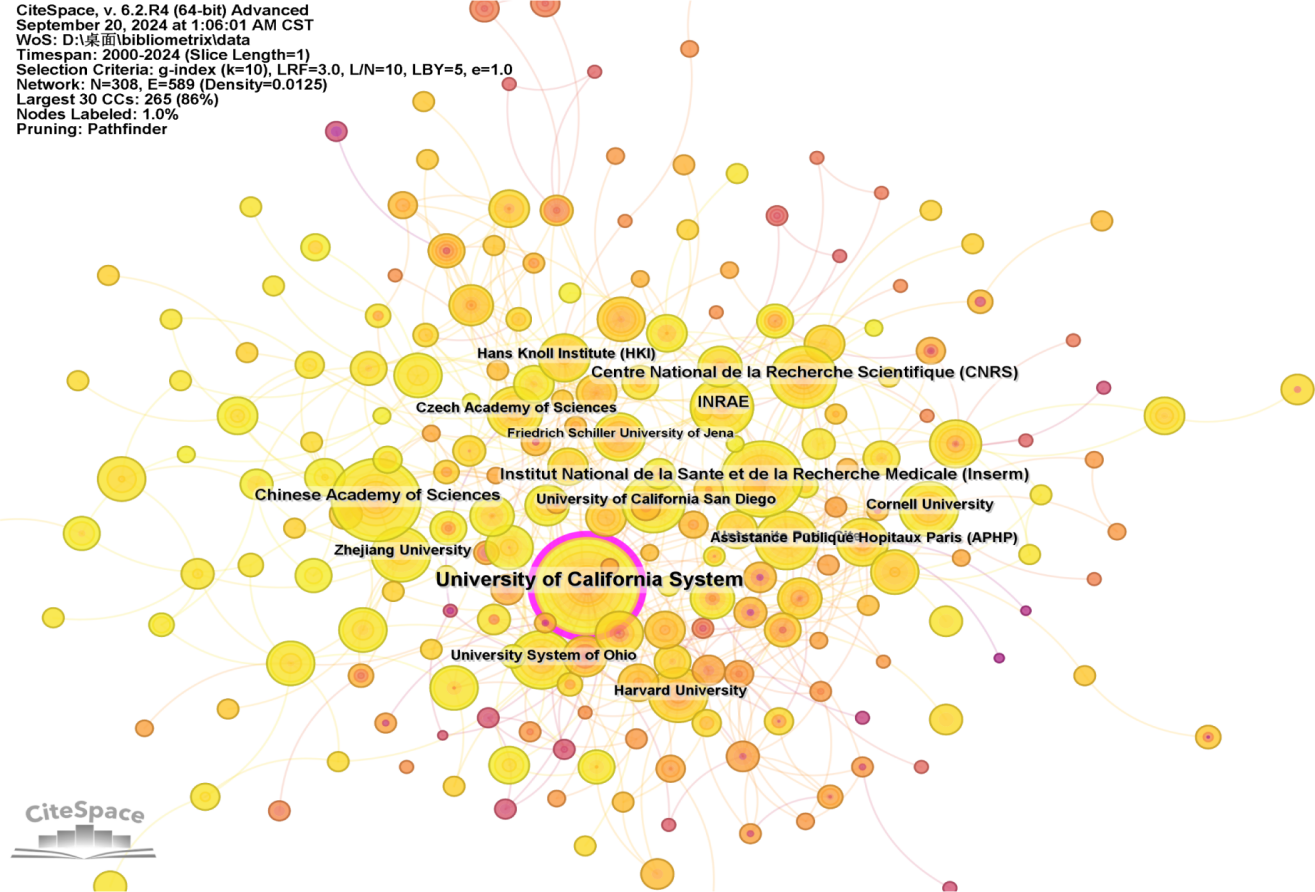
Visualization of the institutions involved in research on intestinal fungal. The study encompassed English-language articles published between 2000 and 2024, with the removal of any duplicate publications.

**Figure 4**, created by CiteSpace, presents an institutional co-occurrence map that illustrates the top-tier institutions engaged in intestinal fungal research. This map underscores their substantial publication contributions and prominence in the field. The institutions are represented as nodes in the visualization, and their relative sizes reflect their impact and significance within the research community. The links between nodes signify collaboration, and the color coding of these links represents different time periods. Nodes that exceed a centrality threshold of 0.1 are identified as central nodes, indicating their importance and significant influence in the study. These nodes are distinguished by purple circles.

### 3.4 Journal of high yield

**Figure 5:**
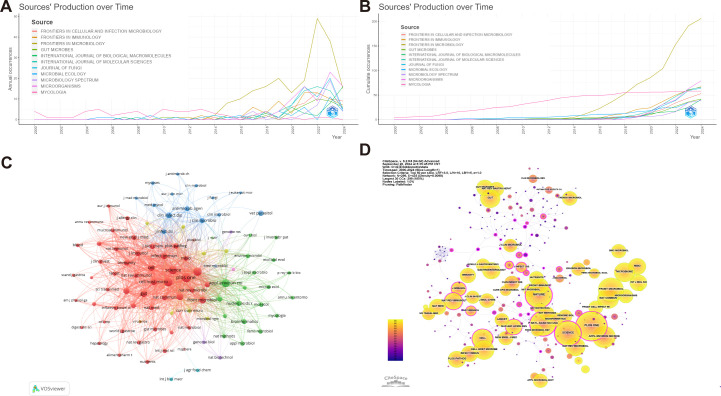
Publications and co-citation network of journals to the field of intestinal fungal research. **(A)** Annual publications of the top 10 journals. **(B)** Cumulative publications of the top 10 journal. **(C)** The network of collaboration among the top 100 co-cited journals. The nodes indicate the number of citations, while the links represent the intensity of cooperation. **(D)** The journals co-citation network. The use of different colors for nodes and links in the visualization represents the chronological occurrence of co-citation relationships.

A total of 1,034 academic journals have published articles pertaining to intestinal fungal research. **Table 3** presents the top 20 journals in terms of publishing articles on intestinal fungal, accounting for 26.25% of the total articles. **Figures 5A**, **B** exhibit the annual and cumulative publications of the journal, respectively. The publication volume of Frontiers In Microbiology has experienced a significant increase in recent years, showing a steep upward trend. It holds the top spot for the highest number of published studies on intestinal fungal, (4.0, Q2) (206). This is followed by Microorganisms (4.1, Q2) (109), and Frontiers In Immunology (5.7, Q1) (74). Sixteen out of the top 20 journals, ranked by publication count, are categorized as Q1 in the Journal Citation Reports (JCR). Furthermore, 35% of these journals possess an Impact Factor (IF) exceeding 5. The Journal of Frontiers In Microbiology stood out with the highest H-index (41) and G-index (71), closely followed by Frontiers In Immunology, which also achieved an H-index of 25 and a G-index of 43. In contrast, Microorganisms ranked second with a lower H-index of 18 and a G-index of 289, suggesting a lack of studies that meet the highest standards of quality in the field.

**Table 3:** The top 20 journals by publication volume in the domain of intestinal fungal research.

Rank	Journal	Counts	Citations	Average citations	H-index	G-index	JCR	IF(2023)
1	Frontiers In Microbiology	206	2439	11.84	41	71	Q1	4
2	Microorganisms	79	1164	14.73	18	28	Q2	4.1
3	Frontiers In Immunology	67	1505	22.46	25	43	Q1	5.7
4	International Journal Of Molecular Sciences	66	1731	26.23	20	44	Q1	4.9
5	Journal Of Fungi	64	720	11.25	16	31	Q1	4.2
6	Mycologia	58	591	10.19	18	32	Q1	2.6
7	Frontiers In Cellular And Infection Microbiology	53	820	15.47	18	38	Q1	4.6
8	Gut Microbes	42	454	10.81	16	34	Q1	12.2
9	International Journal Of Biological Macromolecules	41	616	15.02	13	24	Q1	7.7
10	Microbial Ecology	39	234	6.00	16	31	Q1	3.3
11	Microbiology Spectrum	39	369	9.46	11	21	Q2	3.7
12	Microbiome	37	373	10.08	17	37	Q1	13.8
13	Nutrients	37	1136	30.70	12	24	Q1	4.8
14	Molecules	36	634	17.61	14	34	Q1	4.2
15	Journal Of Ethnopharmacology	32	262	8.19	15	31	Q1	4.8
16	Applied And Environmental Microbiology	31	381	12.29	19	31	Q1	3.9
17	Mbio	31	319	10.29	17	31	Q1	5.1
18	Plos Pathogens	29	269	9.28	22	29	Q1	5.5
19	Bmc Microbiology	27	299	11.07	11	25	Q2	4
20	Food Function	24	429	17.88	11	19	Q1	9.4

**Figure 5C** showcases the Top 100 co-citation journal network, visualized using VOSviewer, and comprises 12 clusters. Among these clusters, the largest one, represented by the red hue, encompasses 49 journals primarily specializing in Medicine and Biology, with a particular emphasis on Immunology and Molecular Sciences. Remarkably, Plos One, Nature, Proceedings of the National Academy of Sciences of the United States of America and Science holds a prominent position within this cluster. The journal publishes an extensive array of fundamental and clinical research, primarily dedicated to exploring the intricate mechanisms that govern changes in intestinal fungi and their profound connections with human pathologies.

The green cluster encompasses journals covering diverse research areas including Microbiology, Bioinformatics, Mycology, and Applied Microbiology. Within the green cluster, Applied and Environmental Microbiology stands as a central figure in the co-citation network, exhibiting both the highest number of quotations and robust co-citation intensity. Its primary research focus is on the complex interaction and relationship between microorganisms and their hosts, encompassing mechanisms of infection, immune responses, and other related fields.

Within the blue cluster, Clinical Infectious Diseases and Antimicrobial Agents and Chemotherapy occupy a prominent position. The cluster mainly focuses on the research and development of antimicrobial agents, medical mycology, the study of antimicrobial resistance mechanisms, and the clinical application of antifungal drugs. The yellow clustering mainly focuses on cellular microbiology and molecular microbiology, focusing on the latest research trends and cutting-edge technologies in the field of microbiology, including the application and promotion of new methods and technologies in the field of microbiology. Key journals within this cluster include Journal of Bacteriology and Trends in Microbiology. The purple cluster represents the field of biotechnology, with the Journal of Genome Biology leading the cluster. These journals have significantly contributed to the advancement of intestinal fungal research by covering the latest advancements in genomics, bioinformatics, and biotechnology, and offering an extensive overview of the cutting-edge technologies and research progress in this field.

Based on the analysis depicted in **Figure 5D**, Jama-Journal of the American Medical Association emerged as the top-ranked journal in terms of centrality, with a value of 0.32, closely followed by Proceedings of the National Academy of Sciences of the United States of America and Nature, both scoring 0.29. These journals demonstrated remarkable centrality, reflecting their substantial influence and importance within the field.

**Figure 6:**
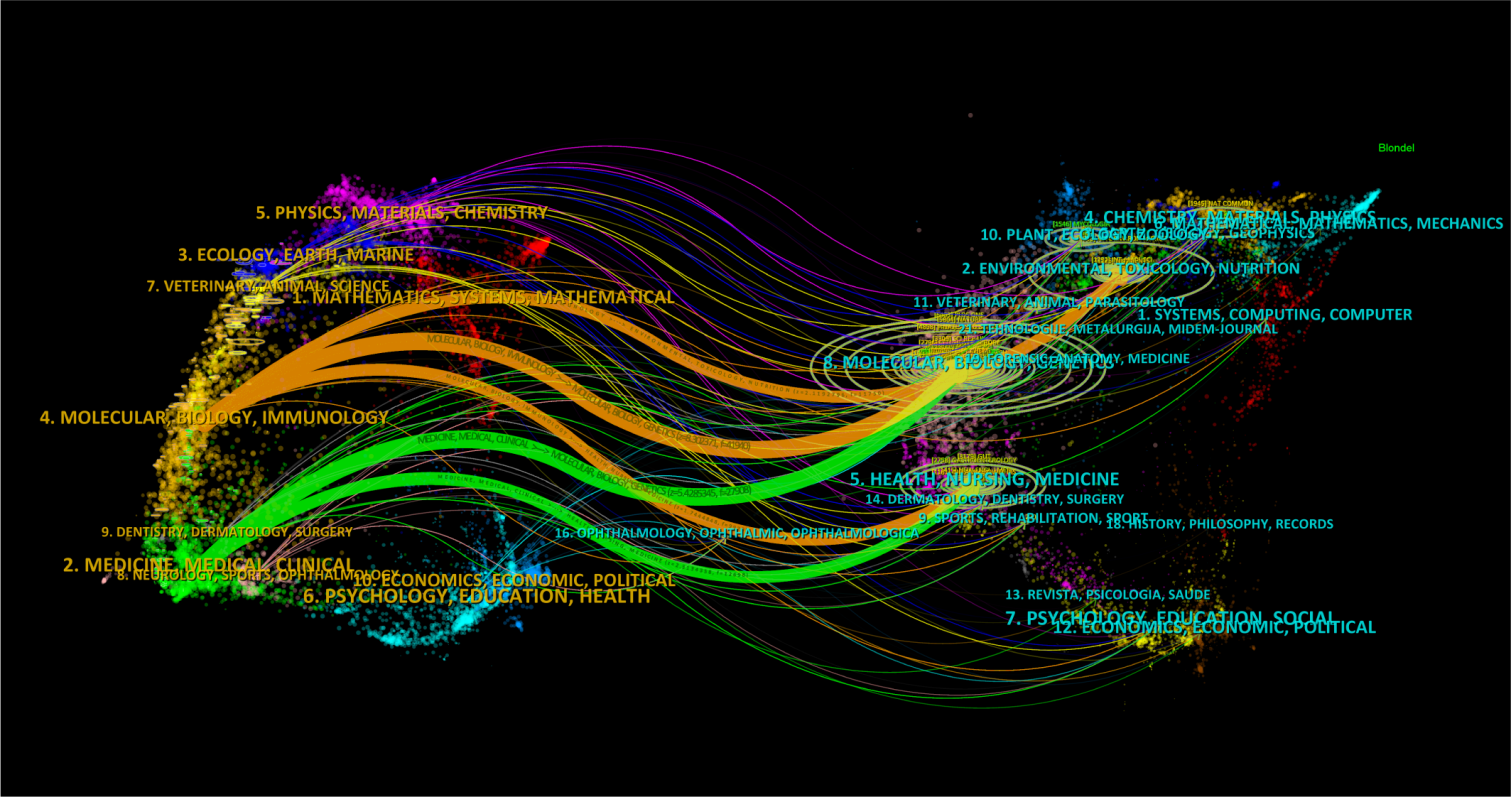
The dual-map visualization presents journals. Citing journals are positioned on the left, while cited journals are positioned on the right. The presence of citation relationships is represented by pathways colored in orange or green.

The dual-map visualization of journals affords profound insights into the distribution of topics, the evolving patterns of citations, and the shifting research priorities among diverse scholarly publications. The labels on the left side of the dual map indicate the journals that are citing, while the labels on the right side indicate the journals being cited. The citation context is visually represented by a colored curve connecting the citing map to the cited map (Chen and Leydesdorff, 2014). Upon analyzing the dual-map overlay depicted in **Figure 6**, two distinct citation paths became evident. These publications were primarily centered on the fields of life sciences and healthcare, encompassing diverse research areas such as molecular, biological, and immunological studies, as well as medicine, medical practice, and clinical domains. Notably, journals spanning multiple disciplines, including molecular biology, genetics, environmental science, toxicology, nutrition, health, nursing, medicine, chemistry, materials science, and physics, showcased articles that were most frequently cited.

### 3.5 Analysis of co-authorship and core authors

A total of 20,559 authors specialize in gut microbiota research. Our study utilizes VOSviewer to analyze their collaboration network, revealing their extensive engagement and cooperation in this field.

**Figure 7:**
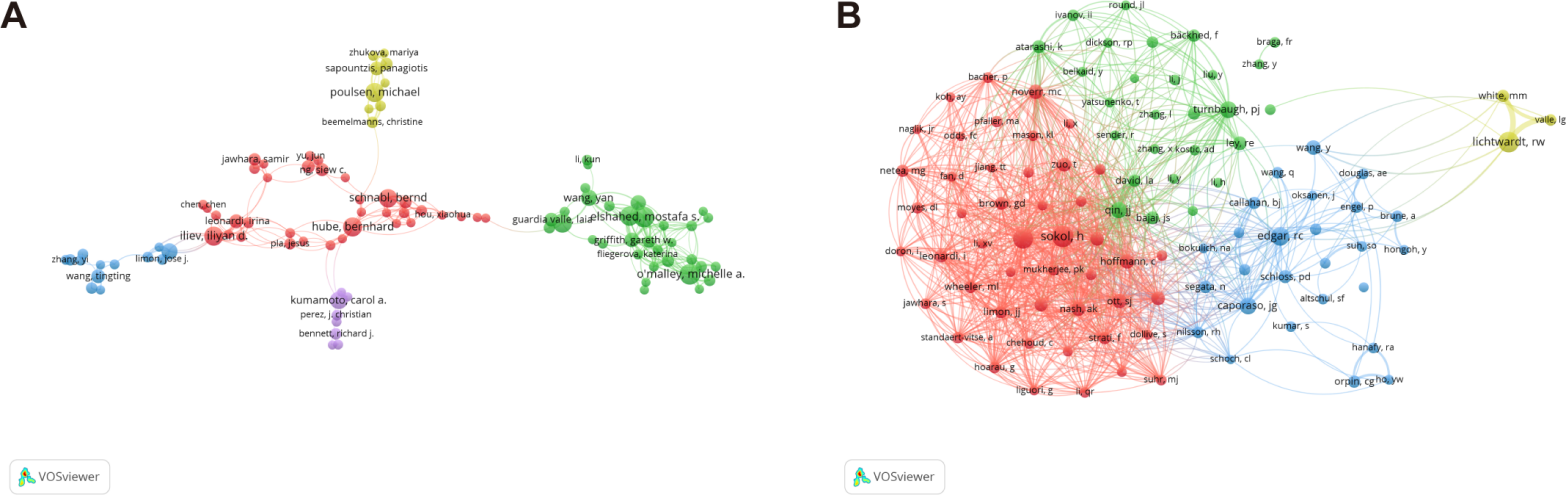
Authors active in the study of intestinal fungus. **(A)** The cooperation network of co-occurring authors visualized using VOSviewer. **(B)** Co-cited authors.

**Table 4** separately lists the top ten authors ranked by their number of publications and citations. **Figure 7A** displays the division of the co-authorship network into five distinct clusters, each uniquely identified by color. Authors are depicted as individual circles, and the lines connecting them represent their collaborative relationships. Different colors were used to indicate distinct clusters of cooperation among authors. The most prolific authors are Mostafa S Elshahed and Michelle A O’Malley, each contributing 23 articles, closely followed by Noha H Youssef and Iliyan D Iliev with 21 articles each, and Bernhard Hube rounds out the top five with 20 articles. The centrality of the author is significantly low, with none of them reaching or exceeding a centrality value of 0.01.

**Table 4:** The top 10 authors by publication and citation count related to intestinal fungal research.

Rank	Authors	Counts	Co-cited author	Citations	Centrality
1	Mostafa S Elshahed	23	Harry Sokol	481	0.03
2	Michelle A O’Malley	23	Iliyan D Iliev	424	0.06
3	Noha H Youssef	21	Peter J Turnbaugh	318	0.04
4	Iliyan D Iliev	21	J Gregory Caporaso	309	0.02
5	Bernhard Hube	20	Wang, Yan	243	0.01
6	Bernd Schnabl	19	Mairi C Noverr	241	0.09
7	Carol A Kumamoto	17	Andrea K Nash	231	0.04
8	Asada Leelahavanichkul	16	Christian Hoffmann	228	0.02
9	Wang, Yan	15	Stephan J Ott	225	0.08
10	David M Underhill	14	Tao Zuo	220	0.01

A total of 103 authors were identified as co-cited when applying a minimum citation threshold of 100 citations per author, demonstrating that they were simultaneously referenced in various publications (**Figure 7B**). Harry Sokol stood out as the leading author, ranking first with a total of 481 citations. Following closely in second place was Iliyan D Iliev., who received 424 citations, while Peter J Turnbaugh took the third spot with 318 citations. Among the co-cited authors, Mairi C. Noverr distinguishes herself with a centrality value of 0.09, holding a substantial lead. Closely following are Stephan J. Ott (0.08) and Iliyan D. Iliev (0.06), both demonstrating a notable degree of influence and interconnectedness within the intricate citation network.

### 3.6 Highly-cited articles

**Table 5** lists the top 10 cited publications. The most cited article was written by Harry Sokol, published in Gut with 227 citations, titled “Fungal microbiota dysbiosis in IBD”. The article underscores the pivotal role of a distinct fungal microbiota dysbiosis in IBD, characterized by alterations in biodiversity and composition. Furthermore, it uncovers disease-specific alterations in the inter-kingdom network, suggesting fungi’s potential role in IBD pathogenesis alongside bacteria (Sokol et al., 2017). Future research should delve into the key players in these interactions and investigate their influence on, as well as their susceptibility to, gut inflammation.

**Table 5:** 10 most cited papers related to the application of human intestinal fungal research.

Rank	Title	Journal	Corresponding author	Affiliation	country	Year	Citations
1	Fungal microbiota dysbiosis in IBD	Gut	Harry Sokol	Sorbonne University	France	2017	227
2	The gut mycobiome of the Human Microbiome Project healthy cohort	Microbiome	Nadim J. Ajami and Joseph F. Petrosino	Baylor College of Medicine	USA	2017	153
3	Reproducible, interactive, scalable and extensible microbiome data science using QIIME 2	Nature Biotechnology	J. Gregory Caporaso	Northern Arizona University	USA	2019	115
4	The gut mycobiota: insights into analysis, environmental interactions and role in gastrointestinal diseases	Nature Reviews Gastroenterology & Hepatology	Harry Sokol	Sorbonne University	France	2019	109
5	Malassezia Is Associated with Crohn’s Disease and Exacerbates Colitis in Mouse Models	Cell Host & Microbe	David M Underhill	Cedars-Sinai Medical Center	USA	2019	102
6	Fungi in the healthy human gastrointestinal tract	Virulence	Mallory J Suhr	University of Nebraska	USA	2017	94
7	Enteric fungal microbiota dysbiosis and ecological alterations in colorectal cancer	Gut	Jun Yu	The Chinese University of Hong Kong	China	2018	89
8	The fungal mycobiome promotes pancreatic oncogenesis via activation of MBL	Nature	George Miller	New York University School of Medicine	USA	2019	89
9	Interactions between commensal fungi and the C-type lectin receptor Dectin-1 influence colitis	Science	David M. Underhill	Cedars-Sinai Medical Center	USA	2012	87
10	Bacteriome and Mycobiome Interactions Underscore Microbial Dysbiosis in Familial Crohn’s Disease	Mbio	M A Ghannoum	Case Western Reserve University	USA	2016	87

**Figure 8:**
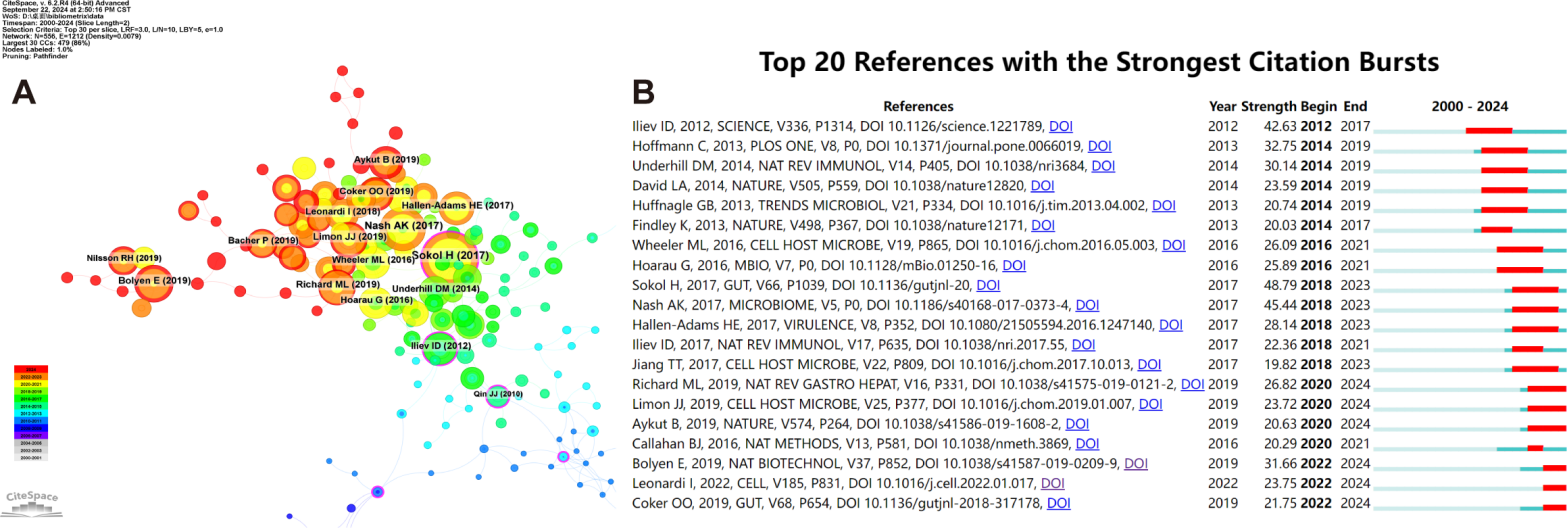
Co-cited references concerning intestinal fungal. **(A)** The visual network of references to work on human intestinal fungus from 2000 through 2024. **(B)** The top 20 references with the strongest citation bursts related to human intestinal fungus between 2000 and 2024. The blue line represents the time from its first appearance to 2024, the red line represents the burst time.

Utilizing the CiteSpace tool, we have obtained key references with high citations in this field (**Figure 8A**). Furthermore, the CiteSpace citation burst functionality enables us to pinpoint references that have garnered significant attention from researchers during a particular time frame (Chen and CiteSpace, 2006). When the burst duration was set to 2 years, a total of 20 references emerged as those with the most prominent citation bursts (**Figure 8B**). Harry Sokol’s seminal article, “Fungal Microbiota Dysbiosis in IBD,” attains a peak citation burst intensity of 48.79, marking a pinnacle in the field (Sokol et al., 2017). Notably, the culmination of six articles, each distinguished by pronounced citation bursts, falls within 2024, underscoring a pronounced and recent surge in academic interest and scrutiny towards these topics (Aykut et al., 2019; Bolyen et al., 2019; Coker et al., 2019; Limon et al., 2019; Richard and Sokol, 2019; Leonardi et al., 2022).

### 3.7 Keyword analysis

**Figure 9:**
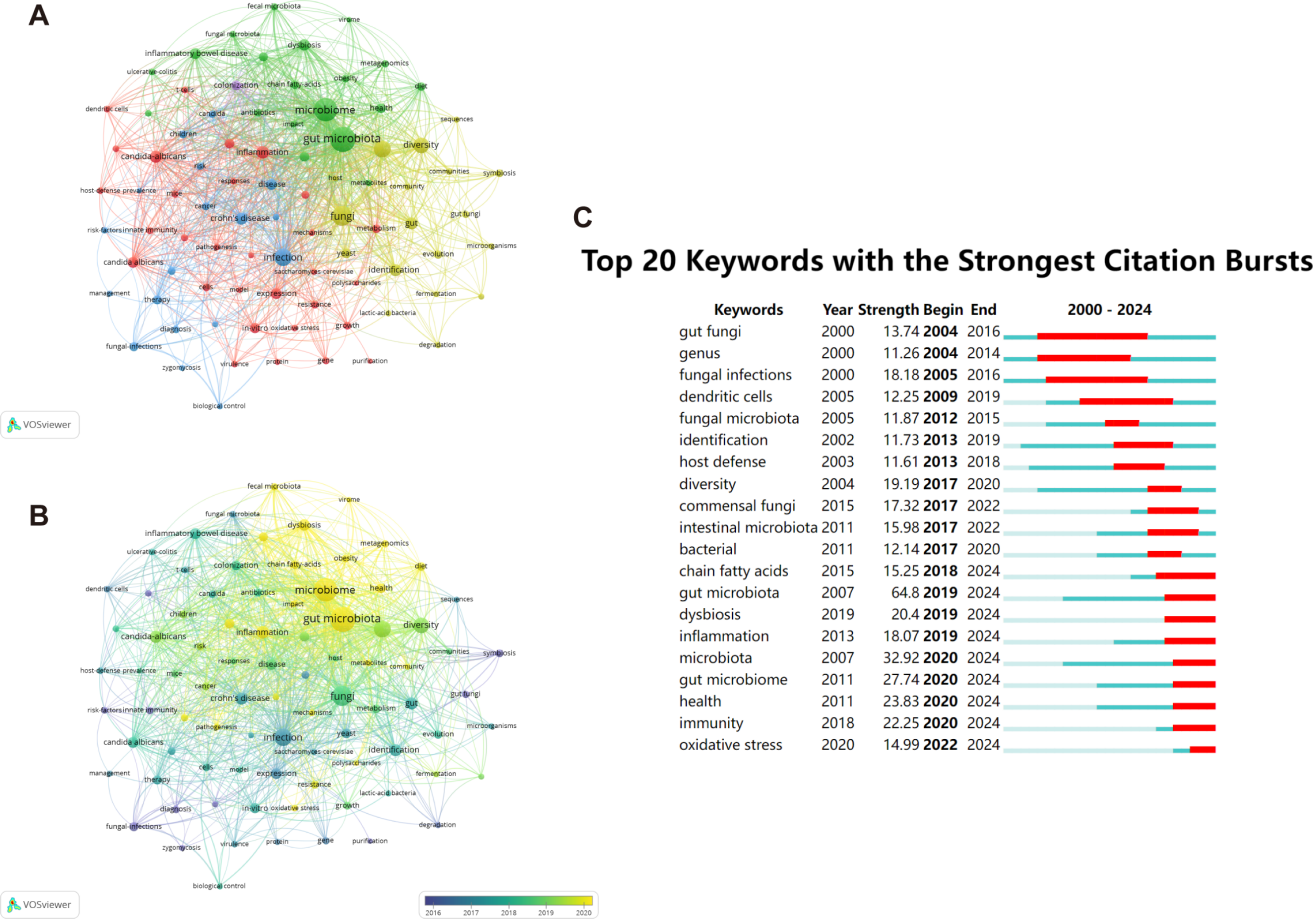
The representation of keyword mapping focusing on intestinal fungal. **(A)** The top 86 keywords were visually represented in a network visualization, with five clusters denoted by different colors. Node size reflects the frequency of occurrence. **(B)** Visualization of keywords based on density. **(C)** Top 20 keywords with the most robust bursts of research on human intestinal fungal from 2000 to 2024.

High-frequency keywords serve as indicators of evolving research frontiers. Utilizing VOSviewer, we pinpointed 16,521 keywords, among which 86 keywords stood out with a minimum frequency of 50 occurrences, emphasizing their importance in the field. There were 5 clusters (**Figure 9A**). “gut microbiota”, “microbiome”, “fungi”, “bacteria”, “infection”, “diversity”, “candida-albicans”, “dysbiosis”, “inflammation”, “crohn’s disease” were the top 10 keywords sorted by frequency of occurrence.

On our overlay visualization map (**Figure 9B**), blue denotes the initial phase of research, whereas yellow represents the more recent period. “risk factors”, “fungal-infections”, “innate immunity”, and “symbiosis” received more attention around 2016. “gut microbiota”, “microbiota”, “health”, and “dysbiosis” have become the foci in recent years. By leveraging the burst keyword detection functionality of CiteSpace, we were able to accurately identify hotspots and trace the evolving research frontiers across various time frames (Chen and CiteSpace, 2006). With the minimum burst duration set to 1, **Figure 9C** presents a visual representation of citation patterns, where red bars indicate keywords that have attracted frequent citations, and blue bars indicate those that have received fewer citations. Among the top 20 keywords exhibiting the most prominent citation bursts, “gut fungi” emerges as the one with the longest burst duration and the highest burst strength, reflecting its significant impact on the field. The field of biomedical research has undergone a profound transition from broad to specific areas of inquiry. Initially, researchers focused on macro-level concepts such as “fungal infections” and their “host defense” mechanisms, examining the direct interactions between pathogens and hosts. However, with the development of sequencing technologies, research has progressively delved into more microscopic and intricate realms, centering on the ecosystem of the “gut microbiome” and its profound impacts on “health,” as well as the specific mechanisms of “oxidative stress” in disease processes. This shift not only exemplifies the integration of interdisciplinary research methodologies but also propels a strategic move from disease treatment towards health prevention.

## 4 Discussion

### 4.1 Global trends in intestinal fungi

Bibliometric analysis is a more and more popular research method, and its spread is attributed to the continuous improvement and accessibility of scientific databases and bibliometric software (Lian et al., 2023). In this study, VOSviewer, CiteSpace, and R software tools were used to investigate the research patterns and main focus areas of gut fungi in the Web of Science Core Collection over the past 24 years. We analyzed a comprehensive data set consisting of 3,954 articles from 3,572 institutions in 117 countries. The participation of 20,559 authors was recorded, and these articles were published in 1,034 journals.

During the period from 2000 to 2024, the annual publication and citation frequency of human gut fungal research significantly increased. This upward trend has become more apparent since 2020, indicating that the scientific community’s interest in gut fungi is increasing and its impact is also growing. Asia, Europe, and North America are leading in research publications on gut fungi, with the United States making outstanding contributions in this field, followed by China and Germany. Among the top ten research institutions in this field, France and the United States have the highest proportion of four each. The University of California System in the United States has the highest h-index, followed closely by Harvard University. The international research scene in this field presently exhibits a notable concentration of collaborations, particularly focused on China and the United States. Overwhelming majority of the top 20 journals in terms of publication volume are Q1 journals. Among the top 20 journals with the highest publication volume, Microbiome has the highest impact factor (IF) of 13.8, establishing its position as a high-quality journal since its establishment in the UK in 2013.

This study offers a diverse array of research findings and intriguing areas of focus, spanning the period from 2000 to 2024. Largely due to advances in high-throughput sequencing technologies, the past decade or two has seen enormous progress in our understanding of the prevalence and diversity of the microbial communities associated with nearly all of our mucosal surfaces. By analyzing the diverse perspectives presented in various publications, keyword clustering, and highly cited articles, we have effectively pinpointed the primary research areas and priorities pertaining to gut fungal communities. These primarily involve the generalization of significant fungal species, and the analysis of the relationships between intestinal fungi and autoimmune diseases, common tumors and their pathogenesis, as well as fungal infectious diseases.

### 4.2 Significant fungal species

#### 4.2.1 Saccharomyces cerevisiae

*Saccharomyces cerevisiae*, a fungus rich in vitamins and protein, holds significant benefits for human health (Barnett, 2000). Its advantageous properties have been evidenced in treating a range of diseases, particularly in enhancing intestinal immune response and strengthening the intestinal barrier (Kelesidis and Pothoulakis, 2012; Pan et al., 2018; Sun et al., 2021). Notably, a deficiency in *S. cerevisiae* can elevate the levels of IL-10, prompting the proposal of its supplementation as an anti-inflammatory approach (Nash et al., 2017). Notably, natural anti-inflammatory effects of *S. cerevisiae* appear to be strain dependent, as some wild *S. cerevisiae* isolates exacerbate colitis (Rinaldi et al., 2013; Chiaro et al., 2017), while others ameliorate it (Tiago et al., 2015).However, despite its potential, *S. cerevisiae* has not yet gained widespread use as a probiotic (Scott et al., 2021).Antibodies against *S. cerevisiae* mannan also called ASCA is another research focus. ASCA IgG and IgA antibodies have been shown to have important diagnostic significance in patients with IBD (Prideaux et al., 2012; Ondrejčáková et al., 2024). Specifically, the presence of ASCA antibodies had a sensitivity of 72% and a specificity of 82% for Crohn’s disease (Linskens et al., 2002; de Vries et al., 2010). However, a growing number of studies have detected high levels of ASCA in patients affected with autoimmune diseases, including antiphospholipid syndrome, systemic lupus erythematosus, type 1 diabetes mellitus, rheumatoid arthritis, spondyloarthritis, and hidradenitis suppurativa (Rinaldi et al., 2013; Maillet et al., 2016; Assan et al., 2020). Recently, increased *S. cerevisiae* levels have been identified in some central nervous system diseases. A study has revealed the presence of elevated serum ASCA levels and an abundance of *S. cerevisiae* in the gut microbiota among patients with *de novo* Parkinson’s disease (Chen et al., 2023). Arnoriaga-­Rodríguez et al. found that subjects with detectable *S. cerevisia* in the gut microbiota showed deficits in executive function and attention (Arnoriaga-Rodríguez et al., 2021), suggesting that *S. cerevisiae* may play a role in the pathogenesis of neurodegenerative disorders. Additionally, increased *S. cerevisiae* levels have also been reported in Autism spectrum disorder (ASD) (Zou et al., 2021).

#### 4.2.2 Candida

*Candida albicans* is a commensal of the mammalian microbiome and the primary pathogenic fungus of humans. It becomes a severe health problem in immunocompromised patients and can cause a wide variety of mucosal and systemic infections (Sardi et al., 2013; Gonzalez-Lara and Ostrosky-Zeichner, 2020). The interaction between *C. albicans* and host cells is characterized by the expression of virulence factors such as adhesins and invasins (Poulain and Jouault, 2004; Hosseini et al., 2019), the secretion of hydrolytic enzymes (Jothi et al., 2021), a transition from yeast to filamentous hyphae form, and the ability to form biofilms (Talapko et al., 2021); these features collectively result in cell adhesion, invasion, and damage. Many studies have shown an interaction between *C. albicans* and gut bacteria (Peleg et al., 2008; Lopez-Medina et al., 2015; Gao et al., 2016; Farrokhi et al., 2021). However, several of the reported interactions are not a single pattern, rather they are both synergistic and antagonistic. Most of the studied bacteria demonstrated both synergistic and antagonistic effects with *C. albicans (* Siavoshi et al., 2005; Bandara et al., 2009; Nash et al., 2016; Graham et al., 2017; Cabral et al., 2018; Uppuluri et al., 2018; Zuo et al., 2018; Eckstein et al., 2020), and just a few bacteria such as *P. aeruginosa (* Morales et al., 2010; Chen et al., 2014), *Salmonella* spp (Winarsih et al., 2019)., and *Lactobacillus* spp (Roy et al., 2014; Graf et al., 2019). demonstrated only antagonism against *C. albicans*, indicating that they are promising as the key to the treatment of *C. albicans* in the future (Dausset et al., 2020).

#### 4.2.3 Malassezia

*Malassezia restricta* constitutes a significant component of the human skin mycobiota and is intricately linked to a diverse array of cutaneous disorders (Vijaya Chandra et al., 2020). In the human gut, the *Malassezia* genus emerges as the second most prevalent fungal genus upon analysis through internal transcribed spacer (ITS) sequencing, with *M. restricta* standing out as the most abundant species within this genus (Suhr et al., 2016; Raimondi et al., 2019). Notably, *M. restricta* plays a pivotal role in the development of gut inflammation and cancer (Spatz and Richard, 2020).Particularly, *M. restricta* is abundantly present in inflammatory bowel disease patients harboring loss-of-function mutations in caspase recruitment domain-containing protein 9 (Card9), a phenomenon that triggers a robust inflammatory response from myeloid phagocytes. Furthermore, colonization of mice with *M. restricta* has been observed to exacerbate the severity of dextran sulfate sodium-induced colitis (Limon et al., 2019).In addition, *Malassezia* species infiltrate pancreatic ductal adenocarcinoma tumors in both humans and mice, contributing to disease progression by activating the complement cascade via mannose-binding lectin (Aykut et al., 2019). Remarkably, a study conducted by Suling Zeng revealed that (Zeng et al., 2023) the presence of *M. restricta* is associated with an increased severity of alcohol-associated liver disease in patients, further substantiating its role in promoting ethanol-induced liver injury in mice.

#### 4.2.4 Other potentially pathogenic fungi

In addition to the aforementioned fungi, potential intestinal pathogens such as *Cryptococcus neoformans* and various *Aspergillus* species (including *A. fumigatus, A. penicillioides, A. niger, and A. flavus*) can pose a significant threat to immunocompromised individuals. *Cryptococcus neoformans*, an opportunistic fungal pathogen, predominantly targets individuals with progressed HIV infections, contributing to 19% of fatalities attributed to AIDS (Botts and Hull, 2010; Rajasingham et al., 2022). The most grave complication arising from cryptococcosis is chronic meningoencephalitis, a condition that frequently proves fatal (Williamson et al., 2017). *A. fumigatus* is the most common *Aspergillus* species that causes invasive aspergillosis (the name given to the vari­ous diseases that are caused by *Aspergillus* spp.). *A. fumigatus*, a causative agent of invasive aspergillosis in immunocompromised individuals, is notorious for its association with an alarmingly high mortality rate, ranging from 30% to 95% (Brown et al., 2012). Individuals who have previously suffered from lung injury are particularly vulnerable to developing chronic pulmonary aspergillosis (CPA) or chronic necrotizing pulmonary aspergillosis (CNPA), stemming from the proliferation of fungal growth within damaged tissues or preexisting cavities. Furthermore, Aspergillus spp. can trigger allergic reactions, manifesting as severe asthma with fungal sensitization (SAFS) or severe allergic bronchopulmonary aspergillosis (ABPA), posing additional health risks.

### 4.3 Gut fungi and autoimmune diseases

#### 4.3.1 IBD

A recent study published in Cell presents a catalog of 760 cultivated gut fungi (CGFs) genomes derived from healthy individuals, emphasizing the taxonomic and functional diversity as well as metabolic potential of human gut fungi (Yan et al., 2024). In addition, the study identifies significant disease-related variations in gut fungal community composition and validates the association between fungal signatures and IBD through animal experiments. In many studies, it has been observed through ITS2 sequencing research that, compared with healthy subjects, the abundance of *Candida* in IBD increases (Chehoud et al., 2015; Lewis et al., 2015), the ratio of Basidiomycota to Ascomycota increases, and there is a strong negative correlation between Basidiomycota and Ascomycota (Nilsson et al., 2006). By analyzing ileal mucosa samples from patients with active Crohn’s disease and healthy individuals, researchers have found that the fungal microbiota in the inflamed mucosa differs significantly from that in non-inflammatory regions (Li et al., 2014). Specifically, the fungal richness and diversity were notably higher in the inflamed mucosa. The predominant fungal species in the inflamed mucosa underwent significant changes, with an increase in the proportions of C*andida* spp., *Gibberella moniliformis*, *Alternaria brassicicola*, and *Cryptococcus neoformans (* Li et al., 2014). The alterations in intestinal fungal microbiota associated with Crohn’s disease are modifiable through treatment with 5-aminosalicylic acid (5-ASA), which results in changes in fungal diversity and composition in the inflamed mucosa and restores the bacterial-fungal correlation (Jun et al., 2020).

Although no specific pathogen or causative agent has been universally identified as the direct cause of IBD, numerous studies provide compelling evidence that fungi play a significant role in driving intestinal inflammation (Caruso et al., 2020). A study revealed genetic diversity in Candida albicans strains colonizing IBD patients’ colonic mucosa. Among them, high damage-causing strains (HD strains) exacerbate intestinal inflammation via IL-1β, reliant on the toxin candidalysin (Li et al., 2022). This highlights strain-specific host-fungal interactions, offering potential diagnostic and therapeutic targets for inflammatory diseases (Li et al., 2022).By comparing the intestinal mucosa of Crohn’s disease (CD) patients with that of healthy controls, it was found that the richness and diversity of fungal communities in inflammatory mucosa were significantly higher than those in non-inflammatory mucosa (Li et al., 2014). The main fungal composition in inflammatory mucosa was characterized by an increase in the proportion of *Candida*, *Cryptococcus*, *Cladosporium*, and *neoformans*, and the study found that the species richness and diversity of fungal communities were associated with the expression of TNF-α, IFN-γ, and IL-10, while fecal fungal community diversity was positively correlated with serum C-reactive protein and CD activity index (Li et al., 2014).In another study (Standaert-Vitse et al., 2009), researchers found that the ASCAs of healthy relatives of patients with familial Crohn’s disease were elevated, which may be caused by changes in the immune response to *Candida albicans*. It is noteworthy that intestinal *Candida albicans* may contribute to inflammation in patients with Crohn’s disease through the IL-23 and T helper 17(Th17) pathway (Zelante et al., 2007).A significant presence of *Candida* colonization is commonly noted among patients suffering from ulcers and IBD (Li et al., 2014; Hoarau et al., 2016; Liguori et al., 2016; Sokol et al., 2017). A study led by Shanghai Jiao Tong University has shown (Yu et al., 2023) that fungal dysbiosis plays a pivotal role in promoting inflammatory bowel disease by strengthening glutaminolysis in CD4+ T cells. Fungi significantly enhance the process of oxidative phosphorylation (OXPHOS) through the augmentation of glutaminolysis. Mechanistically, tje investigation has uncovered that fungi trigger the activation of the dectin-1-Syk-NF-κB signaling pathway, leading to the upregulation of key enzymes and transporters involved in glutaminolysis. Consequently, these findings indicate that targeting fungal interactions in the human gut may emerge as a promising therapeutic approach for inflammatory bowel disease.

#### 4.3.2 SLE

Systemic lupus erythematosus (SLE) is a typical autoimmune disease, which is characterized by the immune system attacking its own tissues and producing a large number of autoantibodies. It is well known that SLE is influenced by genetic and environmental factors. As an important environmental factor, the intestinal microbiota has been found to be correlated with the occurrence and development of SLE (Chen et al., 2021; Zhang L. et al., 2021).Although the mechanism of fungal involvement in the pathogenesis of SLE remains unclear, animal experiments have revealed that *Candida albicans*, which is known to produce intestinal (1 → 3)-β-D-dextran and elevate its serum levels upon colonization in the intestine (Panpetch et al., 2018; Saithong et al., 2021), specifically contributes to an increase in systemic inflammation associated with active lupus. This intestinal translocation of (1 → 3)-β-D-dextran primarily fuels the inflammatory response through the activation of Dectin-1 (Goodridge et al., 2011; Issara-Amphorn et al., 2020).A study has shown that long-term use of antibiotics can increase the infection and overgrowth of *Candida* in the intestine of mice (Dollive et al., 2013). Once SLE patients are diagnosed, they may require lifelong medication, and the application of glucocorticoids and immunosuppressants may result in secondary infections that necessitate anti-infective treatments. These therapeutic factors all impact the characteristics of the intestinal microbiota. Intestinal fungi have also been proven to play a role as opportunistic pathogens in immune-mediated diseases and antibiotic treatments (Gutwinski et al., 2010).

#### 4.3.3 T1D

Type 1 diabetes (T1D) is an immune inflammatory disease caused by the selective destruction of insulin-producing islet cells (β cells) by autoreactive T cells (Thompson et al., 2023).Through a comparative analysis of the fecal fungal composition between T1D patients and healthy controls, utilizing Internal Transcribed Spacer (ITS) sequencing, it was discovered that the abundance of *Saccharomyces* was significantly lower in the T1D group compared to the control group, while the abundances of *Candida*, *Cryptococcus*, *Udeniomyces*, and *Xylodon* increased in T1D patients (Salamon et al., 2021). And the study found that there was a significant negative correlation between the level of high-density lipoprotein cholesterol and *Cladosporium* in T1D diabetic patients, while there was a positive correlation between the levels of total cholesterol and low-density lipoprotein cholesterol and *Saccharomyces cerevisiae*, and alanine aminotransferase was significantly positively correlated with *Cryptococcus* (Salamon et al., 2021). Studies in children have found that the levels of *Bifidobacterium* were lower in patients with type 1 diabetes compared to the control group, whereas the abundance of *Candida albicans* and *Enterobacteriaceae* other than *Escherichia coli* was increased (Soyucen et al., 2014), many studies have confirmed this conclusion (Gosiewski et al., 2014; Gürsoy et al., 2018), possibly due to increased glucose in blood or impaired immune systems (Auchtung et al., 2022). In women with T1D, an increased abundance of yeast during late pregnancy, associated with a decrease in beneficial bacteria such as *Faecalibacterium prausnitzii*, is accompanied by intestinal inflammation and impairment of epithelial integrity (Bandala-Sanchez et al., 2022). By comparing the expression of intestinal bacteria and fungi in children with positive diabetes-related autoantibodies (IAA, GADA, IA-2A, or ICA) with that in autoantibody-negative children with HLA-conferred susceptibility to T1D and following them up for 8 years, fungal ecological imbalance characterized by a large number of yeast and *Candida* in feces was found in some autoantibody-positive children who developed T1D. Studies have confirmed that intestinal symbiotic bacteria interfere with fungal colonization and compete for surfaces and nutrients (Hooper and Macpherson, 2010). Additionally, Short-chain fatty acids (SCFAs) produced by bacteria can inhibit the virulence of Candida by preventing the yeast-hypha transition. Bacteria can also regulate epithelial barrier function and integrity through their SCFA metabolites, such as butyrate (Otte et al., 2009). Therefore, the relatively low abundance of *Clostridium* and butyrate-producing bacteria found in autoantibody-positive children may lead to increased colonization of *Candida*.

### 4.4 Gut fungi and tumors

#### 4.4.1 fungi and common tumors

The alterations in fungal flora in colorectal cancer have garnered significant research attention. A comparative analysis of paired biopsy samples from colorectal adenomas and their adjacent healthy mucosa, involving sequencing of the ITS1 region, revealed a diminished genus-level diversity in adenomas compared to their surrounding tissue (Luan et al., 2015). Both *Phoma* and *Candida* genera were consistently observed in all samples. Furthermore, when adenomas were categorized into advanced and non-advanced stages, noteworthy distinctions were uncovered between the adenoma and adjacent mucosa samples. These disparities were attributed to distinct operational taxonomic units (OTUs), primarily driven by four unidentified taxa that exhibited varying abundances (Luan et al., 2015). An additional analysis encompassing fecal fungi from 131 subjects, including those with CRC, colon polyps, and healthy controls, revealed that ascomycetes and basidiomycetes were the most prevalent fungal groups (Gao et al., 2017). Notably, the ratio of basidiomycetes to ascomycetes in CRC and polyp samples was significantly diminished, contrary to what has been reported in other studies. Intriguingly, the fungal α diversity, assessed using the Shannon and Simpson indices, was observed to be higher in both early and late stages of CRC. By quantitative real-time PCR (qPCR), rather than sequencing-based methods, it was determined that the total fungal load in fecal samples from 87 CRC patients was higher than that in 22 healthy controls. Moreover, among these CRC patients, the proportion of *Candida tropicalis* was higher, while the proportion of *Candida albicans* was lower (Wang et al., 2018).

Multi-queue analysis not only confirms that intestinal fungal ecological imbalance is related to CRC, but also discovers that the difference of fungal species can be used as a diagnostic marker for CRC (Coker et al., 2019).The researchers found that samples clustered separately based on the stage of cancer, while there was no significant difference in alpha diversity between CRC patients and controls. At the species level, 29 species, including *Malassezia globosa* and four Aspergillus species, were enriched in CRC, while nine species, including *Lipomyces starkeyi* and *S. cerevisiae*, were depleted in CRC. Among the expanded fungal species, the carcinogenic aflatoxin-producing *Aspergillus flavus* species displayed the highest enrichment in CRC. This disease-associated fungal signature enabled the distinction of CRC patients from healthy controls after correcting for multiple confounders using linear regression, which was corroborated by ethnically diverse validation cohorts (with an area under the curve (AUC) of 0.82 for the Chinese validation cohort and 0.74 for the European validation cohort). Moreover, the study revealed that even early-stage CRC could be differentiated from healthy samples and validated by the validation cohorts (AUC of 0.81 for the Chinese validation cohort and 0.72 for the European validation cohort), indicating the potential use of the fecal mycobiome signature as a biomarker in the future.

The abnormal colonization of *Candida albicans* and *Malassezia furfur* within the intestine plays a pivotal role in promoting the development of hepatocellular carcinoma (HCC) (Zhang et al., 2023). A thorough examination of fungal alpha-diversity revealed that patients diagnosed with HCC and cirrhosis possess a diminished fungal diversity when compared to healthy individuals (Zhang et al., 2023). It is noteworthy that individuals infected with *C. albicans* and *M. furfur* demonstrated a substantial elevation in tumor weight and volume, whereas there were no notable changes in body weight observed among the other groups (Zhang et al., 2023).

It has been discovered that (Aykut et al., 2019) pancreatic ductal adenocarcinoma (PDAC) exhibits a remarkable ~3000-fold increase in fungal presence when compared to a healthy pancreas, a finding that holds true in both animal models and human studies. Significantly, these fungi demonstrate the ability to migrate from the intestinal lumen into the pancreatic parenchyma. This was further corroborated by Aykut et al (Aykut et al., 2019), who administered GFP-labeled *S. cerevisiae* to mice with tumors via oral gavage and observed that the fungi were able to migrate into the pancreas (Aykut et al., 2019). Recently, Ablam and his team discovered that (Alam et al., 2022) the fungal mycobiome species *Alternaria alternata* is capable of triggering the secretion of IL-33 and the activation of type 2 immunity in pancreatic cancer.

#### 4.4.2 Intratumoral fungi

In 2022, two articles published in the journal CELL sparked a research boom on fungi within tumors (Dohlman et al., 2022; Narunsky-Haziza et al., 2022). Dohlman ‘s study analyzed whole-genome sequencing data from The Cancer Genome Atlas (TCGA) for tumors in the head and neck, esophagus, stomach, colon, rectum, breast, lung, and brain, revealing higher fungal signals in lung and gastrointestinal tumors (except for the esophagus), with the highest fungal load in lower gastrointestinal tumors (Dohlman et al., 2022). *Candida albicans* was the most abundant fungal species in gastrointestinal tumors, and several potentially transiently colonizing fungi, such as *Saccharomyces cerevisiae* and *Cyberlindnera jadinii*, were also detected, possibly originating from dietary sources. In lung tumors, *Blastomyces dermitidis/gilchristii* was enriched, while in breast tumors, several *Malassezia* species and *Yarrowia lipolytica* were more abundant. Inside the tumors, symbiotic communities centered around *Candida albicans* or *Saccharomyces cerevisiae* were particularly prominent. The presence of these communities suggests that these fungi are associated with specific tumor types or states. Tumor cells associated with *Candida albicans* displayed specific transcriptional trends. For instance, compared to head and neck tumors without *Candida albicans*, those containing the fungus showed downregulation in genes related to cell adhesion, tumor suppression, and epithelial-mesenchymal transition (EMT). Furthermore, on one hand, colon tumors containing *Candida albicans* were more prone to metastasis. On the other hand, in gastric tumors with *Candida albicans*, genes related to immune response were upregulated, unlike those without the fungus.

Another study utilized formalin-fixed paraffin-embedded (FFPE) or frozen samples to investigate tumor samples from breast, lung, melanoma, ovary, colon, brain, bone, and pancreas, as well as corresponding normal adjacent tissue controls (Narunsky-Haziza et al., 2022). The study found that fungal loads detected in colon and lung tumors were higher compared to negative controls, while a non-significant trend was observed in breast tumors. To clarify the location of these fungi, the authors employed multiple staining methods and concluded that in pancreatic, breast, and ovarian cancers, fungi were primarily located within cancer cells. However, in melanoma and lung cancer, fungi overlapped with macrophages, suggesting specific interactions between fungi and cell types in different tumor types. Breast cancer exhibited the highest level of fungal-bacterial cross-kingdom co-occurrence, with these fungi mainly clustering around *Aspergillus* or *Malassezia*. This indicates that the presence of fungi in the tumor microenvironment may be related to specific cancer types. To delineate the immune landscapes elicited by fungi in different cancer types, the authors described three distinct fungal-bacterial-immune clusters (termed “mycotypes”), two of which were characterized by higher inflammation and lymphocyte exhaustion, while the third was associated with a robust macrophage response.

#### 4.4.3 Mechanisms of fungal impacts on cancer

The influence of fungi on tumors is primarily mediated through three aspects: 1) the secretion of fungi-induced carcinogenic metabolites, 2) the modulation of the immune system by fungi, and 3) the promotion of tumor metastasis by fungi. The metabolites mediated by fungi primarily include acetaldehyde (Theruvathu et al., 2005), nitrosamines (Hsia et al., 1981), candidalysin (Moyes et al., 2016), aflatoxins (Pickova et al., 2021), and patulin (Singh et al., 2022). Fungi play an indispensable role in the various stages of immune system development, which has been extensively confirmed in other studies (Sohrabi et al., 2010; Wang et al., 2018; Liu et al., 2022). Given the pivotal role of inflammation and defective immune surveillance in cancer, most of the mechanistic relationships discovered between fungi and cancer thus far involve, at the least, a component of the immune system. Research on the role of fungi in promoting tumor metastasis is primarily reflected in the use of a transplanted oral squamous cell carcinoma (OSCC) *in vivo* model (Vadovics et al., 2021), where the oral colonization of *Candida albicans* increases the expression of metastasis-related genes. This could potentially become a hot research topic in the future for exploring the interactions between fungi and tumors.

### 4.5 Infectious diseases caused by gut fungi

Invasive fungal diseases (IFDs) frequently occur as complications among critically ill patients and those suffering from underlying immune system imbalances. In Europe, *candidiasis* and *aspergillosis* were the leading fungal infections, while *mucormycosis* was more prevalent in India (Lass-Flörl and Steixner, 2023). Fungal pathogens pose a significant threat to public health, as timely diagnosis can be challenging, and many fungal species exhibit resistance to currently available antifungal treatments (Arastehfar et al., 2020).

Candidiasis is an opportunistic fungal infection originating from the Ascomycota phylum and the Candida genus, primarily causing superficial fungal diseases such as skin or mucosal infections, as well as invasive or systemic infections (Ahmed et al., 2022). The most prevalent pathogens within the Candida genus are categorized into either I) Candida albicans or II) non-albicans Candida (NAC) infections (Denning, 2023). NAC infections, which encompass *Candida glabrata (Nakaseomyces glabrata)*, *Candida parapsilosis*, *Candida tropicalis*, and *Candida krusei (Pichia kudriavzevii)*, are exhibiting an upward trend. Among these, multidrug-resistant *C. glabrata* and *C. auris* are particularly noteworthy. *C. auris* is an emerging fungus renowned for its persistence, high resistance, and ease of transmission, causing outbreaks in various healthcare settings, thereby posing a significant global health threat (Briano et al., 2022). Over 90% of its strains are resistant to fluconazole, approximately 35% are resistant to amphotericin B, and more than 40% are resistant to two or more antifungal classes (Lockhart et al., 2017).

Aspergillosis refers to an infection caused by opportunistic molds from the family Ascomycetaceae and the genus *Aspergillus (* Chen et al., 2022). *Aspergillus* comprises hundreds of species, some of which are potential pathogens that cause infections, with a mortality rate of up to 50% when treated and 99% when untreated (Sabino et al., 2021). *Aspergillus*-related diseases may manifest as acute invasive aspergillosis (IA), chronic or allergic bronchopulmonary aspergillosis, and/or aspergilloma. As one of the most common causes of invasive fungal infections, IA affects approximately 300,000 patients annually, with approximately 30 million people at risk (Sabino et al., 2021). *Aspergillus fumigatus* accounts for 90% of infections in the genus Aspergillus, followed by *Aspergillus flavus*, *Aspergillus nidulans*, and *Aspergillus terreus (* Binder and Lass-Flörl, 2011). Azole drugs (voriconazole and/or posaconazole) are the first-line treatment and antifungal prophylaxis options, but Aspergillus fumigatus has increasing resistance to azoles (Sabino et al., 2021). A Portuguese study showed that 15.5% of sensitive Aspergillus fumigatus and 41.2% of cryptic Aspergillus fumigatus strains were resistant to azoles (Sabino et al., 2021). In addition, co-infection of *Aspergillus* with viral diseases is very common, and approximately one-third of influenza patients may develop Aspergillus-related infections, making influenza an independent risk factor for IA, with an odds ratio of 5.19 (González-García et al., 2022). The mortality rate of influenza-associated pulmonary aspergillosis (IAPA) ranges from 45% to 61% (Dewi et al., 2021), and studies from Asia emphasize that approximately 28% of severe influenza patients are diagnosed with invasive pulmonary aspergillosis (Huang et al., 2019).

## 5 Strength and limitations

First, due to the constraints imposed by the visualization software on data formats, we exclusively gathered textual data from the WoSCC. Nevertheless, given its widespread usage globally, the WoSCC adequately satisfied our analytical requirements. Second, we narrowed our focus to English publications, specifically original articles and reviews, excluding other types. Third, citation-related metrics are inherently dynamic, evolving over time. Therefore, we must consider a comprehensive range of indices, including the Journal Impact Factor, Source Normalized Impact per Paper, and SCImago Journal Rank, to ensure a holistic analysis. Fourth, while updates to the database may introduce minor variations in our findings, their overall impact is anticipated to be minimal. Furthermore, while the study presents relevant analysis charts from the Scopus database in the **Supplementary Materials**, there are still some important databases that have not been taken into consideration, such as the PubMed database. We believe that a future research comparing three major databases (WoS, Scopus, and PubMed) would be a valuable new direction, potentially offering more comprehensive and insightful findings.

## 6 Conclusion

This study provides an overview of the existing literature on human gut fungi; it conducts a quantitative and qualitative analysis of the major journals, most-cited articles, and most relevant authors in the field of gut fungi publications from 2002 to 2024. Furthermore, the analysis reveals geographical disparities in the publications. The United States and the University of California System emerge as the country and institution with the highest betweenness centrality, respectively. Since 2020, there has been a rapid increase in the number of publications. The United States and China have made the most significant contributions to the field of gut fungi and are leading in international collaboration. In recent years, with the development of gene sequencing technology and the gradual reduction of costs, research hotspots have evolved from gut fungal infections to microbiomics. Researchers are more committed to exploring the interaction mechanisms between gut fungi and human diseases.

## Data Availability

The original contributions presented in the study are included in the article/**Supplementary Material**. Further inquiries can be directed to the corresponding author.

## References

[B1] Abbas A. M. (2012). Bounds and inequalities relating h-index, g-index, e-index and generalized impact factor: an improvement over existing models. PloS One 7, e33699. doi: 10.1371/journal.pone.0033699 22496760 PMC3319552

[B2] Ahmed N. Mahmood M. S. Ullah M. A. Araf Y. Rahaman T. I. Moin A. T.. (2022). COVID-19-associated candidiasis: possible patho-mechanism, predisposing factors, and prevention strategies. Curr. Microbiol. 79, 127. doi: 10.1007/s00284-022-02824-6 35287179 PMC8918595

[B3] Alam A. Levanduski E. Denz P. Villavicencio H. S. Bhatta M. Alhorebi L.. (2022). Fungal mycobiome drives IL-33 secretion and type 2 immunity in pancreatic cancer. Cancer Cell 40, 153–167.e11. doi: 10.1016/j.ccell.2022.01.003 35120601 PMC8847236

[B4] Arastehfar A. Gabaldón T. Garcia-Rubio R. Jenks J. D. Hoenigl M. Salzer H. J.F.. (2020). Drug-resistant fungi: an emerging challenge threatening our limited antifungal armamentarium. Antibiotics (Basel) 9. doi: 10.3390/antibiotics9120877 PMC7764418 33302565

[B5] Arnoriaga-Rodríguez M. Mayneris-Perxachs J. Coll C. Pérez-Brocal V. Ricart W. Moya A.. (2021). Subjects with detectable Saccharomyces cerevisiae in the gut microbiota show deficits in attention and executive function. J. Intern. Med. 290, 740–743. doi: 10.1111/joim.v290.3 34051000

[B6] Assan F. Gottlieb J. Tubach F. Lebbah S. Guigue N. Hickman G.. (2020). Anti-Saccharomyces cerevisiae IgG and IgA antibodies are associated with systemic inflammation and advanced disease in hidradenitis suppurativa. J. Allergy Clin. Immunol. 146, 452–455.e5. doi: 10.1016/j.jaci.2020.01.045 32061710

[B7] Auchtung T. A. Stewart C. J. Smith D. P. Triplett E. W. Agardh D. Hagopian W. A.. (2022). Temporal changes in gastrointestinal fungi and the risk of autoimmunity during early childhood: the TEDDY study. Nat. Commun. 13, 3151. doi: 10.1038/s41467-022-30686-w 35672407 PMC9174155

[B8] Aykut B. Pushalkar S. Chen R. Li Q. Abengozar R. Kim J. I.. (2019). The fungal mycobiome promotes pancreatic oncogenesis via activation of MBL. Nature 574, 264–267. doi: 10.1038/s41586-019-1608-2 31578522 PMC6858566

[B9] Bandala-Sanchez E. Roth-Schulze A. J. Oakey H. Penno M. A.S. Bediaga N. G. Naselli G.. (2022). Women with type 1 diabetes exhibit a progressive increase in gut Saccharomyces cerevisiae in pregnancy associated with evidence of gut inflammation. Diabetes Res. Clin. Pract. 184, 109189. doi: 10.1016/j.diabres.2022.109189 35051423

[B10] Bandara H. Yau J. Y.Y. Watt R. M. Jin L. J. Samaranayake L. P. (2009). Escherichia coli and its lipopolysaccharide modulate in *vitro* Candida biofilm formation. J. Med. Microbiol. 58, 1623–1631. doi: 10.1099/jmm.0.012989-0 19661208

[B11] Barnett J. A. (2000). A history of research on yeasts 2: Louis Pasteur and his contemporaries, 1850-1880. Yeast 16, 755–771. doi: 10.1002/1097-0061(20000615)16:8<755::AID-YEA587>3.0.CO;2-4 10861901

[B12] Binder U. Lass-Flörl C. (2011). Epidemiology of invasive fungal infections in the mediterranean area. Mediterr J. Hematol. Infect. Dis. 3, e20110016. doi: 10.4084/mjhid.2011.016 21625305 PMC3103242

[B13] Bolyen E. Rideout J. R. Dillon M. R. Bokulich N. A. Abnet C. C. Al-Ghalith G. A.. (2019). Reproducible, interactive, scalable and extensible microbiome data science using QIIME 2. Nat. Biotechnol. 37, 852–857. doi: 10.1038/s41587-019-0209-9 31341288 PMC7015180

[B14] Botts M. R. Hull C. M. (2010). Dueling in the lung: how Cryptococcus spores race the host for survival. Curr. Opin. Microbiol. 13, 437–442. doi: 10.1016/j.mib.2010.05.003 20570552 PMC2920366

[B15] Briano F. Magnasco L. Sepulcri C. Dettori S. Dentone C. Mikulska M.. (2022). Candida auris candidemia in critically ill, colonized patients: cumulative incidence and risk factors. Infect. Dis. Ther. 11, 1149–1160. doi: 10.1007/s40121-022-00625-9 35404010 PMC8995918

[B16] Brown G. D. Denning D. W. Gow N. A. Levitz S. M. Netea M. G. White T. C. (2012). Hidden killers: human fungal infections. Sci. Transl. Med. 4, 165rv13. doi: 10.1126/scitranslmed.3004404 23253612

[B17] Cabral D. J. Penumutchu S. Norris C. Morones-Ramirez J. R. Belenky P. (2018). Microbial competition between Escherichia coli and Candida albicans reveals a soluble fungicidal factor. Microb. Cell 5, 249–255. doi: 10.15698/mic2018.05.631 29796389 PMC5961918

[B18] Cancino C. A. Merigo J. M. Coronado F. Dessouky Y. Dessouky M. (2017). Forty years of Computers & Industrial Engineering: A bibliometric analysis. Comput. Ind. Eng. 113, 614–629. doi: 10.1016/j.cie.2017.08.033

[B19] Caruso R. Lo B. C. Núñez G. (2020). Host-microbiota interactions in inflammatory bowel disease. Nat. Rev. Immunol. 20, 411–426. doi: 10.1038/s41577-019-0268-7 32005980

[B20] Chehoud C. Albenberg L. G. Judge C. Hoffmann C. Grunberg S. Bittinger K.. (2015). Fungal signature in the gut microbiota of pediatric patients with inflammatory bowel disease. Inflammation Bowel Dis. 21, 1948–1956. doi: 10.1097/MIB.0000000000000454 PMC4509842 26083617

[B21] Chen A. I. Dolben E. F. Okegbe C. Harty C. E. Golub Y. Thao S.. (2014). Candida albicans ethanol stimulates Pseudomonas aeruginosa WspR-controlled biofilm formation as part of a cyclic relationship involving phenazines. PloS Pathog. 10, e1004480. doi: 10.1371/journal.ppat.1004480 25340349 PMC4207824

[B22] Chen B. D. Jia X. M. Xu J. Y. Zhao L. D. Ji J. Y. Wu B. X.. (2021). An autoimmunogenic and proinflammatory profile defined by the gut microbiota of patients with untreated systemic lupus erythematosus. Arthritis Rheumatol 73, 232–243. doi: 10.1002/art.41511 33124780

[B23] Chen C. Leydesdorff L. (2014). Patterns of connections and movements in dual-map overlays: A new method of publication portfolio analysis. J. Assoc. Inf. Sci. Technol. 65, 334–351. doi: 10.1002/asi.2014.65.issue-2

[B24] Chen C. A. Ho C. H. Wu Y. C. Chen Y. C. Wang J. J. Liao K. M.. (2022). Epidemiology of aspergillosis in cancer patients in Taiwan. Infect. Drug Resist. 15, 3757–3766. doi: 10.2147/IDR.S370967 35859914 PMC9289572

[B25] Chen C. M. CiteSpace I. I. (2006). Detecting and visualizing emerging trends and transient patterns in scientific literature. J. Am. Soc. Inf. Sci. Technol. 57, 359–377. doi: 10.1002/asi.20317

[B26] Chen S. Zhang Y. Dai W. Qi S. Tian W. Gu X.. (2020). Publication trends and hot spots in postoperative cognitive dysfunction research: A 20-year bibliometric analysis. J. Clin. Anesth. 67, 110012. doi: 10.1016/j.jclinane.2020.110012 32942145

[B27] Chen Y. Zhang L. Y. Fang Y. Li C. Xia D. D. Zhang G.. (2023). Elevated serum anti-Saccharomyces cerevisiae antibody accompanied by gut mycobiota dysbiosis as a biomarker of diagnosis in patients with *de novo* Parkinson disease. Eur. J. Neurol. 30, 3462–3470. doi: 10.1111/ene.v30.11 36694359

[B28] Chiaro T. R. Soto R. Zac Stephens W. Kubinak J. L. Petersen C. Gogokhia L.. (2017). A member of the gut mycobiota modulates host purine metabolism exacerbating colitis in mice. Sci. Transl. Med. 9. doi: 10.1126 / scitranslmed aaf9044 10.1126/scitranslmed.aaf9044 PMC5994919 28275154

[B29] Coker O. O. Nakatsu G. Dai R. Z. Wu W. K. K. Wong S. H. Ng S. C.. (2019). Enteric fungal microbiota dysbiosis and ecological alterations in colorectal cancer. Gut 68, 654–662. doi: 10.1136/gutjnl-2018-317178 30472682 PMC6580778

[B30] Dausset C. Bornes S. Miquel S. Kondjoyan N. Angenieux M. Nakusi L.. (2020). Identification of sulfur components enhancing the anti-Candida effect of Lactobacillus rhamnosus Lcr35. Sci. Rep. 10, 17074. doi: 10.1038/s41598-020-74027-7 33051479 PMC7553951

[B31] Denning D. W. (2023). Fungal nomenclature: managing change is the name of the game. Open Forum Infect. Dis. 10, ofad395. doi: 10.1093/ofid/ofad395 37547850 PMC10404000

[B32] de Vries M. van der Horst-Bruinsma I. van Hoogstraten I. van Bodegraven A. von Blomberg A. B. M. Ratnawati H.. (2010). pANCA, ASCA, and OmpC antibodies in patients with ankylosing spondylitis without inflammatory bowel disease. J. Rheumatol 37, 2340–2344. doi: 10.3899/jrheum.100269 20810508

[B33] Dewi I. M. Janssen N. A. Rosati D. Bruno M. Netea M. G. Brüggemann R. J.. (2021). Invasive pulmonary aspergillosis associated with viral pneumonitis. Curr. Opin. Microbiol. 62, 21–27. doi: 10.1016/j.mib.2021.04.006 34034082

[B34] Dohlman A. B. Klug J. Mesko M. Gao I. H. Lipkin S. M. Shen X.. (2022). A pan-cancer mycobiome analysis reveals fungal involvement in gastrointestinal and lung tumors. Cell 185, 3807–3822.e12. doi: 10.1016/j.cell.2022.09.015 36179671 PMC9564002

[B35] Dollive S. Chen Y. Y. Grunberg S. Bittinger K. Hoffmann C. Vandivier L.. (2013). Fungi of the murine gut: episodic variation and proliferation during antibiotic treatment. PloS One 8, e71806. doi: 10.1371/journal.pone.0071806 23977147 PMC3747063

[B36] Eckstein M. T. Moreno-Velásquez S. D. Pérez J. C. (2020). Gut bacteria shape intestinal microhabitats occupied by the fungus candida albicans. Curr. Biol. 30, 4799–4807.e4. doi: 10.1016/j.cub.2020.09.027 33035488

[B37] Farrokhi Y. Al-Shibli B. Al-Hameedawi D. F. Neshati Z. Makhdoumi A. (2021). Escherichia coli enhances the virulence factors of Candida albicans, the cause of vulvovaginal candidiasis, in a dual bacterial/fungal biofilm. Res. Microbiol. 172, 103849. doi: 10.1016/j.resmic.2021.103849 34089837

[B38] Feng H. Chen J. Zhang Z. Lou Y. Zhang S. Yang W. (2023). A bibliometric analysis of artificial intelligence applications in macular edema: exploring research hotspots and Frontiers. Front. Cell Dev. Biol. 11, 1174936. doi: 10.3389/fcell.2023.1174936 37255600 PMC10225517

[B39] Gao Y. Jiang X. Lin D. Chen Y. Tong Z. (2016). The Starvation Resistance and Biofilm Formation of Enterococcus faecalis in Coexistence with Candida albicans, Streptococcus gordonii, Actinomyces viscosus, or Lactobacillus acidophilus. J. Endod. 42, 1233–1238. doi: 10.1016/j.joen.2016.05.002 27316318

[B40] Gao R. Kong C. Li H. Huang L. Qu X. Qin N.. (2017). Dysbiosis signature of mycobiota in colon polyp and colorectal cancer. Eur. J. Clin. Microbiol. Infect. Dis. 36, 2457–2468. doi: 10.1007/s10096-017-3085-6 28821976

[B41] González-García P. Alonso-Sardón M. Rodríguez-Alonso B. Almeida H. Romero-Alegría Á. Vega-Rodríguez V. J.. (2022). How has the aspergillosis case fatality rate changed over the last two decades in Spain? J. Fungi (Basel) 8. doi: 10.3390/jof8060576 PMC9225319 35736059

[B42] Gonzalez-Lara M. F. Ostrosky-Zeichner L. (2020). Invasive candidiasis. Semin. Respir. Crit. Care Med. 41, 3–12. doi: 10.1055/s-0040-1701215 32000280

[B43] Goodridge H. S. Reyes C. N. Becker C. A. Katsumoto T. R. Ma J. Wolf A. J.. (2011). Activation of the innate immune receptor Dectin-1 upon formation of a ‘phagocytic synapse’. Nature 472, 471–475. doi: 10.1038/nature10071 21525931 PMC3084546

[B44] Gosiewski T. Salamon D. Szopa M. Sroka A. Malecki M. T. Bulanda M. (2014). Quantitative evaluation of fungi of the genus Candida in the feces of adult patients with type 1 and 2 diabetes - a pilot study. Gut Pathog. 6, 43. doi: 10.1186/s13099-014-0043-z 25328543 PMC4201707

[B45] Graf K. Last A. Gratz R. Allert S. Linde S. Westermann M.. (2019). Keeping Candida commensal: how lactobacilli antagonize pathogenicity of Candida albicans in an in vitro gut model. Dis. Model. Mech. 12. doi: 10.1242/DM.039719 PMC6765188 31413153

[B46] Graham C. E. Cruz M. R. Garsin D. A. Lorenz M. C. (2017). Enterococcus faecalis bacteriocin EntV inhibits hyphal morphogenesis, biofilm formation, and virulence of Candida albicans. Proc. Natl. Acad. Sci. U.S.A. 114, 4507–4512. doi: 10.1073/pnas.1620432114 28396417 PMC5410809

[B47] Gürsoy S. Koçkar T. Atik SU Önal Z Önal H Adal E. (2018). Autoimmunity and intestinal colonization by Candida albicans in patients with type 1 diabetes at the time of the diagnosis. Korean J. Pediatr. 61, 217–220. doi: 10.3345/kjp.2018.61.7.217 30032588 PMC6106689

[B48] Gutwinski S. Erbe S. Münch C. Janke O. Müller U. Haas J. (2010). Severe cutaneous Candida infection during natalizumab therapy in multiple sclerosis. Neurology 74, 521–523. doi: 10.1212/WNL.0b013e3181cef810 20142621

[B49] Hoarau G. Mukherjee P. K. Gower-Rousseau C. Hager C. Chandra J. Retuerto M. A.. (2016). Bacteriome and mycobiome interactions underscore microbial dysbiosis in familial crohn’s disease. mBio 7. doi: 10.1128/mBio.01250-16 PMC5030358 27651359

[B50] Hooper L. V. Macpherson A. J. (2010). Immune adaptations that maintain homeostasis with the intestinal microbiota. Nat. Rev. Immunol. 10, 159–169. doi: 10.1038/nri2710 20182457

[B51] Hosseini S. S. Ghaemi E. Noroozi A. Niknejad F. (2019). Zinc oxide nanoparticles inhibition of initial adhesion and ALS1 and ALS3 gene expression in candida albicans strains from urinary tract infections. Mycopathologia 184, 261–271. doi: 10.1007/s11046-019-00327-w 30903582

[B52] Hsia C. C. Sun T. T. Wang Y. Y. Anderson L. M. Armstrong D. Good R. A. (1981). Enhancement of formation of the esophageal carcinogen benzylmethylnitrosamine from its precursors by Candida albicans. Proc. Natl. Acad. Sci. U.S.A. 78, 1878–1881. doi: 10.1073/pnas.78.3.1878 7015348 PMC319238

[B53] Hu W. Chen N. Yan W. Pei P. Wei Y. Zhan X. (2022). Knowledge mapping of olfactory dysfunction: A bibliometric study. Front. Syst. Neurosci. 16, 904982. doi: 10.3389/fnsys.2022.904982 35770245 PMC9234575

[B54] Huang L. Zhang N. Huang X. Xiong S. Feng Y. Zhang Y.. (2019). Invasive pulmonary aspergillosis in patients with influenza infection: A retrospective study and review of the literature. Clin. Respir. J. 13, 202–211. doi: 10.1111/crj.2019.13.issue-4 30661296

[B55] Issara-Amphorn J. Chancharoenthana W. Visitchanakun P. Leelahavanichkul A. (2020). Syk inhibitor attenuates polymicrobial sepsis in fcgRIIb-deficient lupus mouse model, the impact of lupus characteristics in sepsis. J. Innate Immun. 12, 461–479. doi: 10.1159/000509111 32927460 PMC7747092

[B56] Jones T. Huggett S. Kamalski J. (2011). Finding a way through the scientific literature: indexes and measures. World Neurosurg. 76, 36–38. doi: 10.1016/j.wneu.2011.01.015 21839937

[B57] Jothi R. Sangavi R. Kumar P. Pandian S. K. Gowrishankar S. (2021). Catechol thwarts virulent dimorphism in Candida albicans and potentiates the antifungal efficacy of azoles and polyenes. Sci. Rep. 11, 21049. doi: 10.1038/s41598-021-00485-2 34702898 PMC8548306

[B58] Jun X. Ning C. Yang S. Zhe W. Na W. Yifan Z.. (2020). Alteration of fungal microbiota after 5-ASA treatment in UC patients. Inflammation Bowel Dis. 26, 380–390. doi: 10.1093/ibd/izz207 31750918

[B59] Kelesidis T. Pothoulakis C. (2012). Efficacy and safety of the probiotic Saccharomyces boulardii for the prevention and therapy of gastrointestinal disorders. Therap. Adv. Gastroenterol. 5 (2), 111–125. doi: 10.1177/1756283X11428502 PMC3296087 22423260

[B60] Lass-Flörl C. Steixner S. (2023). The changing epidemiology of fungal infections. Mol. Aspects Med. 94, 101215. doi: 10.1016/j.mam.2023.101215 37804792

[B61] Leonardi I. Gao I. H. Lin W. Y. Allen M. Li X. V. Fiers W. D.. (2022). Mucosal fungi promote gut barrier function and social behavior via Type 17 immunity. Cell 185, 831–846.e14. doi: 10.1016/j.cell.2022.01.017 35176228 PMC8897247

[B62] Lewis J. D. Chen E. Z. Baldassano R. N. Otley A. R. Griffiths A. M. Lee D.. (2015). Inflammation, antibiotics, and diet as environmental stressors of the gut microbiome in pediatric crohn’s disease. Cell Host Microbe 18, 489–500. doi: 10.1016/j.chom.2015.09.008 26468751 PMC4633303

[B63] Li Q. Wang C. Tang C. He Q. Li N. Li J. (2014). Dysbiosis of gut fungal microbiota is associated with mucosal inflammation in Crohn’s disease. J. Clin. Gastroenterol. 48, 513–523. doi: 10.1097/MCG.0000000000000035 24275714 PMC4059552

[B64] Li X. V. Leonardi I. Putzel G. G. Semon A. Fiers W. D. Kusakabe T.. (2022). Immune regulation by fungal strain diversity in inflammatory bowel disease. Nature 603, 672–678. doi: 10.1038/s41586-022-04502-w 35296857 PMC9166917

[B65] Lian Y. Li X. Lan Y. Li Z. Lin X. Huang J.. (2023). Bibliometric and visual analysis in the field of tea in cancer from 2013 to 2023. Front. Oncol. 13, 1296511. doi: 10.3389/fonc.2023.1296511 38273848 PMC10808711

[B66] Liguori G. Lamas B. Richard M. L. Brandi G. da Costa G. Hoffmann T. W.. (2016). Fungal dysbiosis in mucosa-associated microbiota of crohn’s disease patients. J. Crohns Colitis 10, 296–305. doi: 10.1093/ecco-jcc/jjv209 26574491 PMC4957473

[B67] Limon J. J. Tang J. Li D. Wolf A. J. Michelsen K. S. Funari V.v. (2019). Malassezia is associated with crohn’s disease and exacerbates colitis in mouse models. Cell Host Microbe 25, 377–388.e6. doi: 10.1016/j.chom.2019.01.007 30850233 PMC6417942

[B68] Linskens R. K. Mallant-Hent R. C. Groothuismink Z. M. Bakker-Jonges L. E. van de Merwe J. P. Hooijkaas H.. (2002). Evaluation of serological markers to differentiate between ulcerative colitis and Crohn’s disease: pANCA, ASCA and agglutinating antibodies to anaerobic coccoid rods. Eur. J. Gastroenterol. Hepatol. 14, 1013–1018. doi: 10.1097/00042737-200209000-00013 12352222

[B69] Liu Z. Li Y. Li C. Lei G. Zhou L. Chen X.. (2022). Intestinal candida albicans promotes hepatocarcinogenesis by up-regulating NLRP6. Front. Microbiol. 13, 812771. doi: 10.3389/fmicb.2022.812771 35369462 PMC8964356

[B70] Lockhart S. R. Etienne K. A. Vallabhaneni S. Farooqi J. Chowdhary A. Govender N. P.. (2017). Simultaneous emergence of multidrug-resistant candida auris on 3 continents confirmed by whole-genome sequencing and epidemiological analyses. Clin. Infect. Dis. 64, 134–140. doi: 10.1093/cid/ciw691 27988485 PMC5215215

[B71] Lopez-Medina E. Fan D. Coughlin L. A. Ho E. X. Lamont I. L. Reimmann C.. (2015). Candida albicans inhibits pseudomonas aeruginosa virulence through suppression of pyochelin and pyoverdine biosynthesis. PloS Pathog. 11, e1005129. doi: 10.1371/journal.ppat.1005129 26313907 PMC4552174

[B72] Luan C. Xie L. Yang X. Miao H. Lv N. Zhang R.. (2015). Dysbiosis of fungal microbiota in the intestinal mucosa of patients with colorectal adenomas. Sci. Rep. 5, 7980. doi: 10.1038/srep07980 25613490 PMC4648387

[B73] Ma L. Ma J. Teng M. Li Y. (2022). Visual analysis of colorectal cancer immunotherapy: A bibliometric analysis from 2012 to 2021. Front. Immunol. 13, 843106. doi: 10.3389/fimmu.2022.843106 35432385 PMC9009266

[B74] Maillet J. Ottaviani S. Tubach F. Roy C. Nicaise-Rolland P. Palazzo E.. (2016). Anti-Saccharomyces cerevisiae antibodies (ASCA) in spondyloarthritis: Prevalence and associated phenotype. Joint Bone Spine 83, 665–668. doi: 10.1016/j.jbspin.2015.10.011 26992953

[B75] Manos J. (2022). The human microbiome in disease and pathology. Apmis 130, 690–705. doi: 10.1111/apm.v130.12 35393656 PMC9790345

[B76] Morales D. K. Jacobs N. J. Rajamani S. Krishnamurthy M. Cubillos-Ruiz J. R. Hogan D. A. (2010). Antifungal mechanisms by which a novel Pseudomonas aeruginosa phenazine toxin kills Candida albicans in biofilms. Mol. Microbiol. 78, 1379–1392. doi: 10.1111/j.1365-2958.2010.07414.x 21143312 PMC3828654

[B77] Moyes D. L. Wilson D. Richardson J. P. Mogavero S. Tang S. X. Wernecke J.. (2016). Candidalysin is a fungal peptide toxin critical for mucosal infection. Nature 532, 64–68. doi: 10.1038/nature17625 27027296 PMC4851236

[B78] Narunsky-Haziza L. Sepich-Poore G. D. Livyatan I. Asraf O. Martino C. Nejman C. D.. (2022). Pan-cancer analyses reveal cancer-type-specific fungal ecologies and bacteriome interactions. Cell 185, 3789–3806.e17. doi: 10.1016/j.cell.2022.09.005 36179670 PMC9567272

[B79] Nash E. E. Peters B. M. Fidel P. L. Noverr M. C. (2016). Morphology-Independent Virulence of Candida Species during Polymicrobial Intra-abdominal Infections with Staphylococcus aureus. Infect. Immun. 84, 90–98. doi: 10.1128/IAI.01059-15 26483410 PMC4694008

[B80] Nash A. K. Auchtung T. A. Wong M. C. Smith D. P. Gesell J. R. Ross M. C.. (2017). The gut mycobiome of the Human Microbiome Project healthy cohort. Microbiome 5, 153. doi: 10.1186/s40168-017-0373-4 29178920 PMC5702186

[B81] Nilsson R. H. Ryberg M. Kristiansson E. Abarenkov K. Larsson K. H. Kõljalg U. (2006). Taxonomic reliability of DNA sequences in public sequence databases: a fungal perspective. PloS One 1, e59. doi: 10.1371/journal.pone.0000059 17183689 PMC1762357

[B82] Ondrejčáková L. Gregova M. Bubova K. Šenolt L. Pavelka K. (2024). Serum biomarkers and their relationship to axial spondyloarthritis associated with inflammatory bowel diseases. Autoimmun Rev. 23, 103512. doi: 10.1016/j.autrev.2023.103512 38168574

[B83] Otte J. M. Zdebik A. E. Brand S. Chromik A. M. Strauss S. Schmitz F.. (2009). Effects of the cathelicidin LL-37 on intestinal epithelial barrier integrity. Regul. Pept. 156, 104–117. doi: 10.1016/j.regpep.2009.03.009 19328825

[B84] Pan S. Jia B. Liu H. Wang Z. Chai M. Z. Ding M. Z.. (2018). Endogenous lycopene improves ethanol production under acetic acid stress in Saccharomyces cerevisiae. Biotechnol. Biofuels 11, 107. doi: 10.1186/s13068-018-1107-y 29643937 PMC5891932

[B85] Panpetch W. Somboonna N. Bulan D. E. Issara-Amphorn J. Worasilchai N. Finkelman M.. (2018). Gastrointestinal colonization of candida albicans increases serum (1→3)-β-D-glucan, without candidemia, and worsens cecal ligation and puncture sepsis in murine model. Shock 49, 62–70. doi: 10.1097/SHK.0000000000000896 28498297

[B86] Pappas P. G. Lionakis M. S. Arendrup M. C. Ostrosky-Zeichner L. Kullberg B. J. (2018). Invasive candidiasis. Nat. Rev. Dis. Primers 4, 18026. doi: 10.1038/nrdp.2018.26 29749387

[B87] Paterson M. J. Oh S. Underhill D. M. (2017). Host-microbe interactions: commensal fungi in the gut. Curr. Opin. Microbiol. 40, 131–137. doi: 10.1016/j.mib.2017.11.012 29175338 PMC5733715

[B88] Peleg A. Y. Tampakakis E. Fuchs B. B. Eliopoulos G. M. Moellering R. C. Jr. Mylonakis E. (2008). Prokaryote-eukaryote interactions identified by using Caenorhabditis elegans. Proc. Natl. Acad. Sci. U.S.A. 105, 14585–14590. doi: 10.1073/pnas.0805048105 18794525 PMC2567192

[B89] Pickova D. Ostry V. Toman J. Malir F. (2021). Aflatoxins: history, significant milestones, recent data on their toxicity and ways to mitigation. Toxins (Basel) 13. doi: 10.3390/toxins13060399 PMC8227755 34205163

[B90] Poulain D. Jouault T. (2004). Candida albicans cell wall glycans, host receptors and responses: elements for a decisive crosstalk. Curr. Opin. Microbiol. 7, 342–349. doi: 10.1016/j.mib.2004.06.011 15358252

[B91] Prideaux L. De Cruz P. Ng S. C. Kamm M. A. (2012). Serological antibodies in inflammatory bowel disease: a systematic review. Inflammation Bowel Dis. 18, 1340–1355. doi: 10.1002/ibd.21903 22069240

[B92] Raimondi S. Amaretti A. Gozzoli C. Simone M. Righini L. Candeliere F.. (2019). Longitudinal survey of fungi in the human gut: ITS profiling, phenotyping, and colonization. Front. Microbiol. 10, 1575. doi: 10.3389/fmicb.2019.01575 31354669 PMC6636193

[B93] Rajasingham R. Govender N. P. Jordan A. Loyse A. Shroufi A. Denning D. W.. (2022). The global burden of HIV-associated cryptococcal infection in adults in 2020: a modelling analysis. Lancet Infect. Dis. 22, 1748–1755. doi: 10.1016/S1473-3099(22)00499-6 36049486 PMC9701154

[B94] Richard M. L. Sokol H. (2019). The gut mycobiota: insights into analysis, environmental interactions and role in gastrointestinal diseases. Nat. Rev. Gastroenterol. Hepatol. 16, 331–345. doi: 10.1038/s41575-019-0121-2 30824884

[B95] Rinaldi M. Perricone R. Blank M. Perricone C. Shoenfeld Y. (2013). Anti-Saccharomyces cerevisiae autoantibodies in autoimmune diseases: from bread baking to autoimmunity. Clin. Rev. Allergy Immunol. 45, 152–161. doi: 10.1007/s12016-012-8344-9 23292495

[B96] Roldan-Valadez E. Salazar-Ruiz S. Y. Ibarra-Contreras R. Rios C. (2019). Current concepts on bibliometrics: a brief review about impact factor, Eigenfactor score, CiteScore, SCImago Journal Rank, Source-Normalised Impact per Paper, H-index, and alternative metrics. Ir J. Med. Sci. 188, 939–951. doi: 10.1007/s11845-018-1936-5 30511320

[B97] Roy A. Chaudhuri J. Sarkar D. Ghosh P. Chakraborty S. (2014). Role of enteric supplementation of probiotics on late-onset sepsis by candida species in preterm low birth weight neonates: A randomized, double blind, placebo-controlled trial. N Am. J. Med. Sci. 6, 50–57. doi: 10.4103/1947-2714.125870 24678479 PMC3938875

[B98] Sabino R. Gonçalves P. Martins Melo A. Simões D. Oliveira M. Francisco M.. (2021). Trends on aspergillus epidemiology-perspectives from a national reference laboratory surveillance program. J. Fungi (Basel) 7. doi: 10.3390/jof7010028 PMC7825284 33418997

[B99] Saithong S. Saisorn W. Visitchanakun P. Sae-Khow K. Chiewchengchol D. Leelahavanichkul A. (2021). A synergy between endotoxin and (1→3)-beta-D-glucan enhanced neutrophil extracellular traps in candida administered dextran sulfate solution induced colitis in fcGRIIB-/- lupus mice, an impact of intestinal fungi in lupus. J. Inflammation Res. 14, 2333–2352. doi: 10.2147/JIR.S305225 PMC8179808 34103965

[B100] Salamon D. Sroka-Oleksiak A. Gurgul A. Arent Z. Szopa M. Bulanda M.. (2021). Analysis of the gut mycobiome in adult patients with type 1 and type 2 diabetes using next-generation sequencing (NGS) with increased sensitivity-pilot study. Nutrients 13. doi: 10.3390/nu13041066 PMC8064496 33806027

[B101] Sardi J. C. O. Scorzoni L. Bernardi T. Fusco-Almeida A. M. Mendes Giannini M. J. S. (2013). Candida species: current epidemiology, pathogenicity, biofilm formation, natural antifungal products and new therapeutic options. J. Med. Microbiol. 62, 10–24. doi: 10.1099/jmm.0.045054-0 23180477

[B102] Scott B. M. Gutiérrez-Vazquez C. Sanmarco L. M. da Silva Pereira J. A. Li Z. Plasencia A.. (2021). Self-tunable engineered yeast probiotics for the treatment of inflammatory bowel disease. Nat. Med. 27, 1212–1222. doi: 10.1038/s41591-021-01390-x 34183837

[B103] Siavoshi F. Salmanian A. H. Akbari F. Malekzadeh R. Massarrat S. (2005). Detection of Helicobacter pylori-specific genes in the oral yeast. Helicobacter 10, 318–322. doi: 10.1111/j.1523-5378.2005.00319.x 16104948

[B104] Singh N. Dev I. Pal S. Yadav S. K. Idris M. M. Ansari K. M. (2022). Transcriptomic and proteomic insights into patulin mycotoxin-induced cancer-like phenotypes in normal intestinal epithelial cells. Mol. Cell Biochem. 477, 1405–1416. doi: 10.1007/s11010-022-04387-3 35150386

[B105] Sohrabi N. Hassan Z. M. Khosravi A. R. Tebianian M. Mahdavi M. Tootian Z. (2010). Invasive aspergillosis promotes tumor growth and severity in a tumor-bearing mouse model. Can. J. Microbiol. 56, 771–776. doi: 10.1139/W10-064 20921987

[B106] Sokol H. Leducq V. Aschard H. Pham H. P. Jegou S. Landman C. (2017). Fungal microbiota dysbiosis in IBD. Gut 66, 1039–1048. doi: 10.1136/gutjnl-2015-310746 26843508 PMC5532459

[B107] Song L. Liang J. Wang W. Gao J. Chai H. Tan Y.. (2023). Global Trends in Research of Mitochondrial Biogenesis over past 20 Years: A Bibliometric Analysis. Oxid. Med. Cell Longev 2023, 7291284. doi: 10.1155/2023/7291284 36644577 PMC9833928

[B108] Soverini M. Turroni S. Biagi E. Brigidi P. Candela M. Rampelli S. (2019). HumanMycobiomeScan: a new bioinformatics tool for the characterization of the fungal fraction in metagenomic samples. BMC Genomics 20, 496. doi: 10.1186/s12864-019-5883-y 31202277 PMC6570844

[B109] Soyucen E. Gulcan A. Aktuglu-Zeybek A. C. Onal H. Kiykim E. Aydin A. (2014). Differences in the gut microbiota of healthy children and those with type 1 diabetes. Pediatr. Int. 56, 336–343. doi: 10.1111/ped.2014.56.issue-3 24475780

[B110] Spatz M. Richard M. L. (2020). Overview of the potential role of malassezia in gut health and disease. Front. Cell Infect. Microbiol. 10, 201. doi: 10.3389/fcimb.2020.00201 32528901 PMC7265801

[B111] Standaert-Vitse A. Sendid B. Joossens M. François N. Vandewalle-El Khoury P. Branche J.. (2009). Candida albicans colonization and ASCA in familial Crohn’s disease. Am. J. Gastroenterol. 104, 1745–1753. doi: 10.1038/ajg.2009.225 19471251

[B112] Suhr M. J. Banjara N. Hallen-Adams H. E. (2016). Sequence-based methods for detecting and evaluating the human gut mycobiome. Lett. Appl. Microbiol. 62, 209–215. doi: 10.1111/lam.12539 26669281

[B113] Sun S. Xu X. Liang L. Wang X. Bai X. Zhu L.. (2021). Lactic Acid-Producing Probiotic Saccharomyces cerevisiae Attenuates Ulcerative Colitis via Suppressing Macrophage Pyroptosis and Modulating Gut Microbiota. Front. Immunol. 12, 777665. doi: 10.3389/fimmu.2021.777665 34899735 PMC8652295

[B114] Talapko J. Juzbašić M. Matijević T. Pustijanac E. Bekić S. Kotris I.. (2021). Candida albicans-the virulence factors and clinical manifestations of infection. J. Fungi (Basel) 7. doi: 10.3390/jof7020079 PMC7912069 33499276

[B115] Theruvathu J. A. Jaruga P. Nath R. G. Dizdaroglu M. Brooks P. J. (2005). Polyamines stimulate the formation of mutagenic 1,N2-propanodeoxyguanosine adducts from acetaldehyde. Nucleic Acids Res. 33, 3513–3520. doi: 10.1093/nar/gki661 15972793 PMC1156964

[B116] Thompson P. J. Pipella J. Rutter G. A. Gaisano H. Y. Santamaria P. (2023). Islet autoimmunity in human type 1 diabetes: initiation and progression from the perspective of the beta cell. Diabetologia 66, 1971–1982. doi: 10.1007/s00125-023-05970-z 37488322 PMC10542715

[B117] Tiago F. C. Porto B. A. Ribeiro N. S. Moreira L. M. Arantes R. M. Vieira A. T.. (2015). Effect of Saccharomyces cerevisiae strain UFMG A-905 in experimental model of inflammatory bowel disease. Benef Microbes 6, 807–815. doi: 10.3920/BM2015.0018 26322540

[B118] Uppuluri P. Lin L. Alqarihi A. Luo G. Youssef E. G. Alkhazraji S.. (2018). The Hyr1 protein from the fungus Candida albicans is a cross kingdom immunotherapeutic target for Acinetobacter bacterial infection. PloS Pathog. 14, e1007056. doi: 10.1371/journal.ppat.1007056 29746596 PMC5963808

[B119] Vadovics M. Ho J. Igaz N. Alföldi R. Rakk D. Veres É.. (2021). Candida albicans enhances the progression of oral squamous cell carcinoma *in vitro* and *in vivo* . mBio 13, e0314421. doi: 10.1128/mBio.03144-21 35089096 PMC8725587

[B120] van Eck N. J. Waltman L. (2017). Citation-based clustering of publications using CitNetExplorer and VOSviewer. Scientometrics 111, 1053–1070. doi: 10.1007/s11192-017-2300-7 28490825 PMC5400793

[B121] Vijaya Chandra S. H. Srinivas R. Dawson T. L. Jr. Common J. E. (2020). Cutaneous malassezia: commensal, pathogen, or protector? Front. Cell Infect. Microbiol. 10, 614446. doi: 10.3389/fcimb.2020.614446 33575223 PMC7870721

[B122] Wang T. Fan C. Yao A. Xu X. Zheng G. You Y.. (2018). The adaptor protein CARD9 protects against colon cancer by restricting mycobiota-mediated expansion of myeloid-derived suppressor cells. Immunity 49, 504–514.e4. doi: 10.1016/j.immuni.2018.08.018 30231984 PMC6880241

[B123] Williamson P. R. Jarvis J. N. Panackal A. A. Fisher M. C. Molloy S. F. Loyse A.. (2017). Cryptococcal meningitis: epidemiology, immunology, diagnosis and therapy. Nat. Rev. Neurol. 13, 13–24. doi: 10.1038/nrneurol.2016.167 27886201

[B124] Winarsih S. Kosasih T. Putera M. A. Rahmadhiani N. Poernomo E. L. Runtuk K. S.. (2019). β-Glucan of candida albicans cell wall extract inhibits salmonella typhimurium colonization by potentiating cellular immunity (CD8 + and CD4 + T cells). Rev. Soc. Bras. Med. Trop. 52, e20180254. doi: 10.1590/0037-8682-0254-2018 30726315

[B125] Yan Q. Li S. Yan Q. Huo X. Wang C. Wang X.. (2024). A genomic compendium of cultivated human gut fungi characterizes the gut mycobiome and its relevance to common diseases. Cell. 187 (12), 2969-89.e24. doi: 10.1016/j.cell.2024.04.043 38776919

[B126] Yu M. Ding H. Gong S. Luo Y. Lin H. Mu Y.. (2023). Fungal dysbiosis facilitates inflammatory bowel disease by enhancing CD4+ T cell glutaminolysis. Front. Cell Infect. Microbiol. 13, 1140757. doi: 10.3389/fcimb.2023.1140757 37124046 PMC10140311

[B127] Zelante T. De Luca A. Bonifazi P. Montagnoli C. Bozza S. Moretti S.. (2007). IL-23 and the Th17 pathway promote inflammation and impair antifungal immune resistance. Eur. J. Immunol. 37, 2695–2706. doi: 10.1002/eji.200737409 17899546

[B128] Zeng S. Hartmann P. Park M. Duan Y. Lang S. Llorente C.. (2023). Malassezia restricta promotes alcohol-induced liver injury. Hepatol. Commun. 7, e0029. doi: 10.1097/HC9.0000000000000029 36706195 PMC9988279

[B129] Zhang F. Zuo T. Yeoh Y. K. Cheng F. W. T. Liu Q. Tang W.. (2021). *Longitudinal dynamics of gut bacteriome, mycobiome and virome after fecal microbiota transplantation in graft-ve*rsus-host disease. Nat. Commun. 12, 65. doi: 10.1038/s41467-020-20240-x 33397897 PMC7782528

[B130] Zhang L. Qing P. Yang H. Wu Y. Liu Y. Luo Y. (2021). Gut microbiome and metabolites in systemic lupus erythematosus: link, mechanisms and intervention. Front. Immunol. 12, 686501. doi: 10.3389/fimmu.2021.686501 34335588 PMC8319742

[B131] Zhang L. Chen C. Chai D. Li C. Qiu Z. Kuang T.. (2023). Characterization of the intestinal fungal microbiome in patients with hepatocellular carcinoma. J. Transl. Med. 21, 126. doi: 10.1186/s12967-023-03940-y 36793057 PMC9933289

[B132] Zou R. Wang Y. Duan M. Guo M. Zhang Q. Zheng H. (2021). Dysbiosis of gut fungal microbiota in children with autism spectrum disorders. J. Autism Dev. Disord. 51, 267–275. doi: 10.1007/s10803-020-04543-y 32447559

[B133] Zuo T. Wong S. H. Cheung C. P. Lam K. Lui R. Cheung K.. (2018). Gut fungal dysbiosis correlates with reduced efficacy of fecal microbiota transplantation in Clostridium difficile infection. Nat. Commun. 9, 3663. doi: 10.1038/s41467-018-06103-6 30202057 PMC6131390

