# Supplementary material for: Global research trends and hotspots on human intestinal fungi and health: a bibliometric visualization study
Source: Front Cell Infect Microbiol. 2024 Oct 17;14:1460570. doi: 10.3389/fcimb.2024.1460570 (PMC11525014; doi:10.3389/fcimb.2024.1460570)
Supplement: Supplementary file 1 [file DataSheet1.pdf]

Supplement Table 1: The Top 10 Countries with the Most Publications on Gut Fungi in the Scopus Database

Rank	Countries/ regions	counts	citations	Average citations	H-index	Centrality
1	UNITED STATES	794	47564	59.90	209	0.41
2	CHINA	597	10299	17.25	89	0.07
3	INDIA	188	5274	28.05	66	0.06
4	ITALY	161	8354	51.89	97	0.17
5	FRANCE	151	9661	63.98	119	0.13
6	GERMANY	149	9996	67.09	81	0.1
7	UNITED KINGDOM	144	5937	41.23	78	0.18
8	CANADA	111	4026	36.27	66	0.11
9	BRAZIL	106	2763	26.07	49	0.05
10	SPAIN	96	1717	17.89	54	0.04

Supplement Table 2: The Top 10 Institutions by Volume of Research on Intestinal Fungal Studies in the Scopus Database

Rank	Institutions	Country	Counts	H-index	Centrality
1	University Of California	United States	85	41	0.07
2	Harvard Medical School	United States	50	27	0.06
3	University Of California San Diego	United States	47	22	0.01
4	University Of Minnesota	United States	47	21	0
5	Shiraz University Of Medical Sciences	Iran	44	10	0
6	Chulalongkorn University	Thailand	43	21	0
7	University Of Calgary	Canada	43	19	0
8	Mahidol University	Thailand	41	14	0
9	University Of Toronto	Canada	41	22	0
10	The Chinese University Of Hong Kong	China	39	13	0

Supplement Table 3: The top 20 journals by publication volume in the domain of intestinal fungal research in the Scopus

Rank	Journal	Counts	H-index	G-index	JCR	IF(2023)
1	Frontiers In Microbiology	171	37	65	Q1	4
2	Frontiers In Immunology	71	29	47	Q1	5.7
3	Frontiers In Cellular And Infection Microbiology	59	19	35	Q1	4.6
4	Medical Mycology	52	19	33	Q2	2.7
5	Microorganisms	51	14	25	Q2	4.1
6	Journal Of Fungi	50	16	31	Q1	4.2
7	Gut Microbes	43	16	36	Q1	12.2
8	Nutrients	42	13	28	Q1	4.8
9	Microbiology Spectrum	41	11	20	Q2	3.7
10	Mycoses	40	19	32	Q1	4.1
11	World Journal Of Gastroenterology	38	18	38	Q1	4.3
12	Parasitology Research	34	17	25	Q1	1.8
13	Microbiome	29	16	29	Q1	13.8
14	Bmc Infectious Diseases	25	14	25	Q2	3.4
15	Bmc Microbiology	24	11	24	Q2	4
16	Microbial Pathogenesis	24	13	23	Q1	3.3
17	Clinical Infectious Diseases	23	18	23	Q1	8.2
18	Gastroenterology	23	20	23	Q1	25.7
19	Pathogens	23	10	17	Q2	3.3
20	Journal Of Medical Microbiology	22	13	22	Q2	2.4

Supplemen Table 4: 10 most cited papers related to the application of human intestinal fungal research in the Scopus database

Rank	Title	Journal	Corresponding author	Affiliation	country	Year	Citations
1	Gut microbiota and IBD: causation or correlation?	Nature Reviews Gastroenterology & Hepatology	Vesselin T Tomov	University of Pennsylvania	USA	2017	1100
2	Novel perspectives on mucormycosis: pathophysiology, presentation, and management	Clinical Microbiology Reviews	Ashraf Ibrahim	Los Angeles Biomedical Institute at Harbor-UCLA Medical Center	USA	2005	1009
3	ESCMID* guideline for the diagnosis and management of Candida diseases 2012: non-neutropenic adult patients	Clinical Microbiology And Infection	A J Ullmann	University of Cologne	Germany	2012	995
4	Epidemiology and clinical manifestations of mucormycosis	Clinical Infectious Diseases	Dimitrios P Kontoyiannis	National and Kapodistrian University of Athens	Greece	2012	945
5	Fungal microbiota dysbiosis in IBD	Gut	Laurent Beaugerie	Saint Antoine Hospital	France	2017	893
6	Candida infections of medical devices	Clinical Microbiology Reviews	Rabih O Darouiche	Veterans Affairs Medical Center	USA	2004	881
7	Microbial culturomics: paradigm shift in the human gut microbiome study	Clinical Microbiology And Infection	D Raoult	Aix Marseille Université	France	2012	867
8	Invasive candidiasis	Nature Reviews Disease Primers	Bart Jan Kullberg	University of Alabama at Birmingham	USA	2018	700
9	Effect of antimicrobial agents on the ecological balance of human microflora	Lancet Infectious Diseases	C E Nord	Huddinge University Hospital	Sweden	2001	642
10	The epidemiology and clinical manifestations of mucormycosis: a systematic review and meta-analysis of case reports	Clinical Microbiology And Infection	S C-A Chen	Monash University	Australia	2019	593

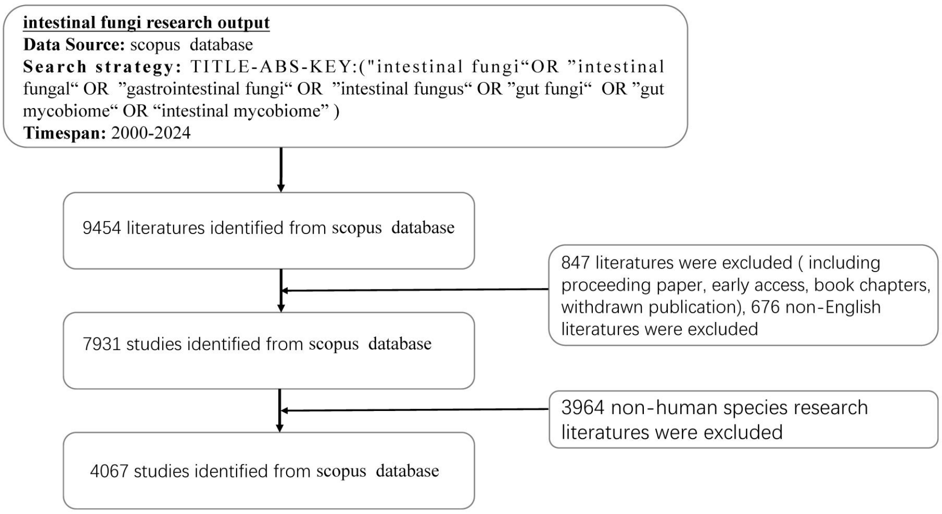

Supplement Figure 1： The diagram illustrates the sequential steps of evaluation and selection in the Scopus database.

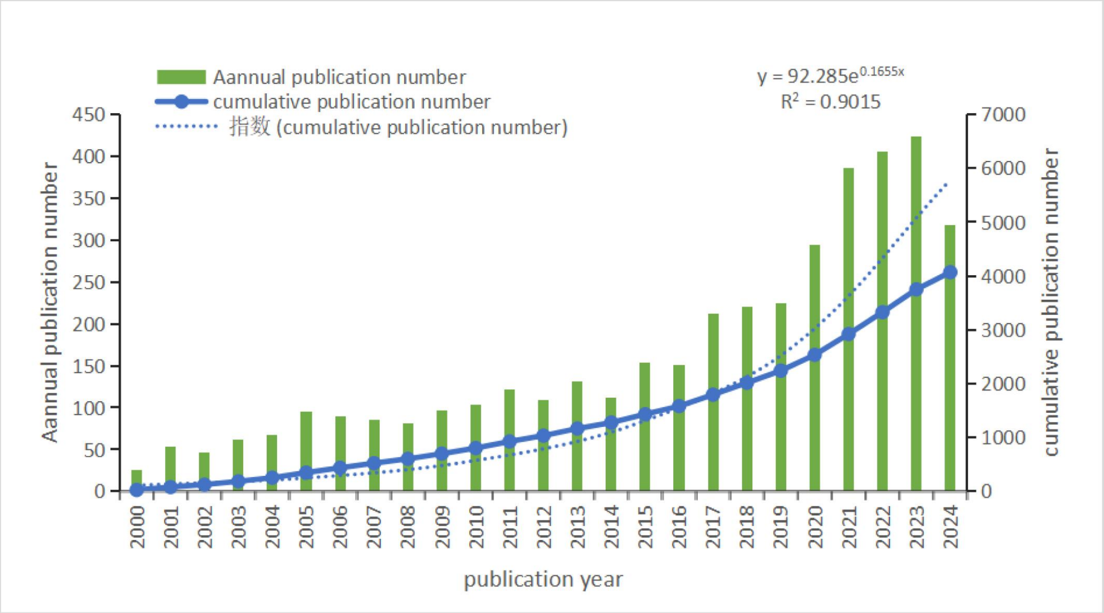

Supplement Figure 2： Overview of Intestinal Fungal-Related Publication Volume in Scopus Database: Annual and Cumulative Publication Counts from 2000 to 2024.

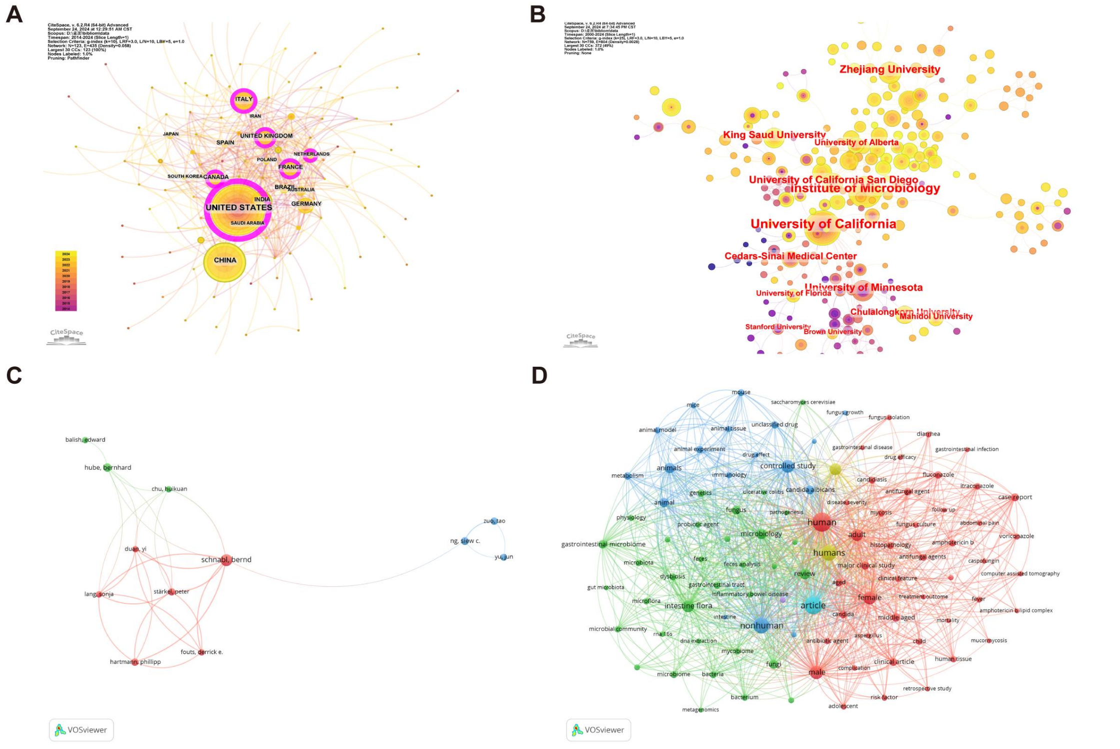

Supplement Figure 3： Co-occurrence Visualization of Intestinal Fungal Research in Scopus Database. **(A)**Visualization of the countries involved in research on intestinal fungal. **(B)** Visualization of the institutions involved in research on intestinal fungal. **(C)** The cooperation network of co-occurring authors visualized using VOSviewer. **(D)** The cooperation network of co-occurring keywords visualized using VOSviewer.

A

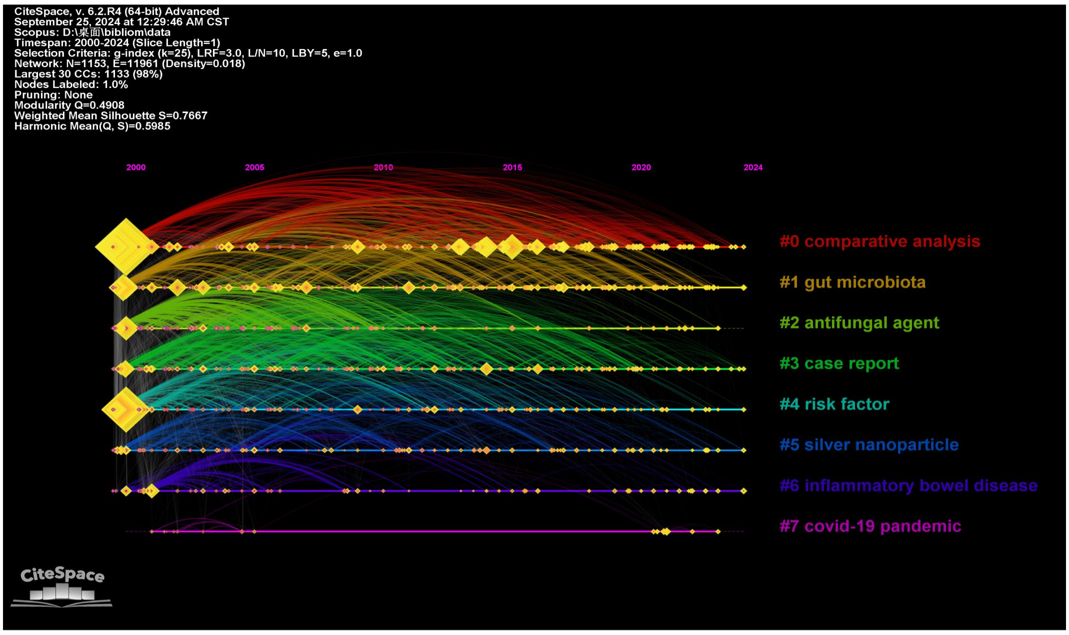

B

Top 20 Keywords with the Strongest Citation Bursts

Keywords	Year	Strength	Begin	End	2000 - 2024
priority journal	2000	103.56	**2000**	2015	
clinical trial	2000	59.56	**2000**	2010	
fluconazole	2000	42.09	**2000**	2013	
candidiasis	2000	41.33	**2000**	2015	
itraconazole	2000	41.25	**2000**	2011	
drug efficacy	2000	39.64	**2000**	2012	
clinical feature	2000	33.22	**2000**	2009	
treatment outcome	2000	30.42	**2000**	2015	
ketoconazole	2000	29.84	**2000**	2010	
amphotericin b	2000	29.37	**2000**	2011	
side effect	2003	33.81	**2003**	2012	
isolation and purification	2001	45	**2014**	2020	
immunology	2000	34.23	**2014**	2019	
drug effects	2014	33.03	**2014**	2017	
pathology	2007	28.78	**2014**	2020	
microbiology	2000	49.43	**2015**	2019	
drug effect	2000	32.46	**2018**	2020	
mycobiome	2014	47.99	**2021**	2024	
dna extraction	2016	44.32	**2022**	2024	
microbial diversity	2014	32.48	**2022**	2024	

Supplement Figure 4： Keyword Clustering and Burst Analysis of Intestinal Fungi Research in Scopus Database. **(A)** Timeline of Keyword Clustering; **(B)** Top 20 Keyword Bursts.
